# Development and Validation of Models to Predict Poor Health-Related Quality of Life Among Adult Survivors of Childhood Cancer

**DOI:** 10.1001/jamanetworkopen.2022.27225

**Published:** 2022-08-17

**Authors:** Fiona Schulte, Yan Chen, Yutaka Yasui, Maritza E. Ruiz, Wendy Leisenring, Todd M. Gibson, Paul C. Nathan, Kevin C. Oeffinger, Melissa M. Hudson, Gregory T. Armstrong, Leslie L. Robison, Kevin R. Krull, I-Chan Huang

**Affiliations:** 1 Department of Oncology, University of Calgary, Calgary, Alberta, Canada; 2 Department of Public Health Sciences, University of Alberta, Edmonton, Alberta, Canada; 3 Department of Epidemiology and Cancer Control, St Jude Children’s Research Hospital, Memphis, Tennessee; 4 Department of Pediatric Hematology/Oncology, MemorialCare Miller Children’s & Women’s Hospital Long Beach,Long Beach, California; 5 Clinical Research Division, Fred Hutchinson Cancer Research Center, Seattle, Washington; 6 Division of Cancer Epidemiology and Genetics, National Cancer Institute, Rockville, Maryland; 7 Division of Haematology/Oncology, The Hospital for Sick Children, Toronto, Ontario, Canada; 8 Department of Medicine, Duke University, Durham, North Carolina; 9 Department of Oncology, St Jude Children’s Research Hospital, Memphis, Tennessee; 10 Department of Psychology, St Jude Children’s Research Hospital, Memphis, Tennessee

## Abstract

**Importance:**

Risk prediction models are important to identify survivors of childhood cancer who are at risk of experiencing poor health-related quality of life (HRQOL) as they age.

**Objective:**

To develop and validate prediction models for a decline in HRQOL among adult survivors of childhood cancer.

**Designs, Setting, and Participants:**

This prognostic study included 4755 adults from the Childhood Cancer Survivor Study (CCSS) diagnosed between January 5, 1970, and December 31, 1986, who completed baseline (time 0 [November 3, 1992, to August 28, 2003]) and 2 follow-up (time 1 [February 12, 2002, to May 21, 2005] and time 2 [January 6, 2014, to November 30, 2016]) surveys. Data were analyzed from June 19, 2019, to February 2, 2022.

**Exposures:**

Sociodemographic, lifestyle, and emotional factors, and chronic health conditions (CHCs) were assessed at time 0 and time 1, and neurocognitive factors were assessed at time 1 to predict HRQOL at time 2 and a decline in HRQOL between time 1 and time 2. Impaired health states were defined as CHC grades 2 to 4 using the modified Common Terminology Criteria for Adverse Events, version 4.03, and mental and neurocognitive status as 1 SD or more below reference levels.

**Main Outcomes and Measures:**

Health-related quality of life was operationalized using the Medical Outcomes Study 36-Item Short Form Health Survey Physical (PCS) and Mental (MCS) Component Summary and classified by optimal (≥40) or suboptimal (<40) at each point (main outcome). A decline in HRQOL was defined as a change from optimal to suboptimal between time 1 and time 2. Multivariable logistic regression identified factors associated with HRQOL decline. The cohort was randomly split into training (80%) and test (20%) data sets for model development and validation; the area under the receiver operating characteristic curve was used to evaluate prediction performance.

**Results:**

A total of 4755 adults (mean [SD] age at time 0, 24.3 [7.6] years; 2623 [55.2%] women) were included in the analysis. Between time 1 and time 2, 285 of 3294 survivors (8.7%) had declining PCS and 278 of 3294 (8.4%) had declining MCS. Risk factors associated with PCS decline included female sex (odds ratio [OR], 1.67 [95% CI, 1.25-2.24]), family income less than $20 000 vs $80 000 or more (OR, 2.00 [95% CI, 1.21-3.30]), presence of CHCs (OR for neurological, 2.16 [95% CI, 1.51-3.10]; OR for endocrine, 2.25 [95% CI, 1.44-3.52]; OR for gastrointestinal tract, 1.89 [95% CI, 1.32-2.69]; OR for respiratory, 1.66 [95% CI, 1.06-2.59]; OR for cardiovascular, 1.53 [95% CI, 1.14-2.06]), and depression (OR, 1.79 [95% CI, 1.20-2.67]). Risk factors associated with MCS decline included unemployment vs full-time employment (OR, 1.68; [95% CI, 1.19-2.38]), current vs never cigarette smoking (OR, 2.03 [95% CI, 1.37-3.00]), depression (OR, 4.29 [95% CI, 2.44-7.55]), somatization (OR, 1.63 [95% CI, 1.05-2.53]), impaired task efficiency (OR, 1.90 [95% CI, 1.34-2.68]), and impaired organization (OR, 1.67 [95% CI, 1.12-2.48]). The areas under the receiver operating characteristic curve for the test models were 0.74 (95% CI, 0.67-0.81) for declining PCS and 0.68 (95% CI, 0.60-0.75) for declining MCS.

**Conclusions and Relevance:**

In this prognostic study of adult survivors of childhood cancer who experienced declining HRQOL, CHCs were associated with a decline in physical HRQOL, whereas current smoking and emotional and neurocognitive impairment were associated with a decline in mental HRQOL. These findings suggest that interventions targeting modifiable risk factors are needed to prevent poor HRQOL in this population.

## Key Points

**Question:**

What is the validity of prediction models that use sociodemographic, lifestyle, and health state factors to predict health-related quality of life (HRQOL) among adult survivors of childhood cancer?

**Findings:**

In this prognostic study of 4755 adult survivors of childhood cancer, chronic health conditions were associated with a decline in physical HRQOL, whereas current cigarette smoking and emotional and neurocognitive impairment were associated with a decline in mental HRQOL. Performance based on the area under receiver operator characteristic curve was 0.74 for declining physical HRQOL and 0.68 for declining mental HRQOL.

**Meaning:**

These findings may help guide clinicians to screen for risk factors during follow-up appointments and provide interventions that target these risk factors to prevent poor HRQOL among adult survivors of childhood cancer.

## Teaser

This prognostic study identifies sociodemographic, lifestyle, and health state factors associated with declining health-related quality of life and examines the performance of models developed to predict these associations among adult survivors of childhood cancer.

## Introduction

The 5-year survival rate for childhood cancer has increased to more than 85%,^1^ resulting in an estimated 500 000 survivors living in the US in 2020.^2^ However, this progress is tempered by adverse sequalae^3^ and premature mortality^4,5^ caused by cancer and its treatment. Indeed, by 50 years of age, nearly 100% of survivors develop chronic health conditions (CHCs), many of which are disabling or life-threatening.^6^ Therefore, a subset of childhood cancer survivors experience poorer health-related quality of life (HRQOL) compared with national norms and/or sibling controls.^7,8^

Health-related quality of life is a critical health metric because it focuses on the effects of health on an individual’s well-being and ability to function within society. It is multidimensional and captures subjective perceptions of physical, psychological, and social aspects of health.^9^ For cancer survivors, assessment of HRQOL allows for the quantification of perceived functioning.^10^ Although previous studies^7,11^ have investigated poor HRQOL among survivors of childhood cancer, a crucial gap in the literature is the ability to predict which survivors might experience poor HRQOL or a decline in HRQOL after many years of treatment. This information is needed to develop interventions that will protect the most vulnerable cancer survivors.

Our understanding of HRQOL among survivors of childhood cancer and the risk factors for poor HRQOL has been limited by methodological designs. Most studies assessing HRQOL have relied on cross-sectional designs, prohibiting the prediction of future HRQOL.^7,11,12^ Although a few longitudinal studies examining HRQOL exist,^13,14^ this work has largely focused on patients undergoing treatment for cancer and the immediate posttherapy period. Cross-sectional studies have revealed sociodemographic factors (eg, female sex), clinical and treatment factors (eg, cranial radiation therapy), depression and neurocognitive deficits,^15^ and health behaviors (eg, smoking)^16,17^ to be associated with poor HRQOL. Given the nature of these study designs, it has been difficult to distinguish the directionality of these associations.

Longitudinal data collected from the Childhood Cancer Survivor Study (CCSS) provide the opportunity to identify risk factors for suboptimal and declining HRQOL that can be targeted in future interventions during follow-up care appointments. Therefore, the aims of this study were (1) to describe the prevalence of future suboptimal and declining HRQOL over time; (2) to identify sociodemographic, lifestyle, and health state factors associated with future suboptimal and declining HRQOL; and (3) to examine the performance of models developed to predict suboptimal and declining HRQOL.

## Methods

### Study Design

This study adhered to the Strengthening the Reporting of Observational Studies in Epidemiology (STROBE) reporting guideline for cohort studies. The CCSS is a multi-institutional longitudinal cohort study of adult patients diagnosed with and treated for childhood cancer at younger than 21 years of age at 26 institutions in the US and Canada between January 5, 1970, and December 31, 1986, and who have 5 or more years of survival.^18,19^ Data collected from the CCSS baseline (time 0: November 3, 1992, through August 28, 2003) and 2 subsequent follow-up (time 1: February 12, 2002, to May 21, 2005; time 2: January 6, 2014, to November 30, 2016) surveys were used in this study. The CCSS was approved by the institutional review board at each treating institution; all participants provided written informed consent. The mean (SD) interval for time 0 to time 1 was 7.7 (1.2) years; for time 1 to time 2, 11.6 (0.7) years.

### Participants and Data Collection

Study participants were adult survivors of childhood cancer from the CCSS. Inclusion criteria consisted of survival of 5 years after diagnosis before 21 years of age. Among eligible participants who completed the time 0 and 1 surveys (n = 9284), we excluded 3290 who did not complete the time 2 survey and 1239 whose surveys were completed by proxies or had missing HRQOL data, leaving 4755 included in the analyses (eFigure in the Supplement). Compared with survivors who had completed the time 1 survey only, those who completed both the time 1 and time 2 surveys were more likely to be women (2623 of 4755 [55.2%] vs 1622 of 3612 [44.9%]) and non-Hispanic White (4333 of 4738 [91.5%] vs 3123 of 3612 [86.5%]); to have a college graduate or postgraduate educational level (1473 of 4601 [32.0%] vs 1272 of 3612 [35.2%]); and to have a higher annual household income (≥$80 000, 1484 of 4755 [31.2%] vs 688 of 3612 [19.0%]). In addition, participants who completed times 1 and 2 surveys were more likely to have nonimpaired emotional distress and neurocognitive functional status but to have some CHCs (*P* < .05; eTable 1 in the Supplement) and to have optimal HRQOL (*P* < .05; eTable 2 in the Supplement). Cancer diagnosis and treatment data were abstracted from medical records at each treating institution.

### Measurement

#### HRQOL Outcomes

We measured HRQOL experienced during the prior 4 weeks at time 1 and time 2 using the Medical Outcomes Study 36-Item Short Form Health Survey. The 36-Item Short Form Health Survey captures 8 domains of HRQOL (physical functioning, role limitations resulting from physical health problems, bodily pain, general health perceptions, vitality, social functioning, role limitations resulting from emotional problems, and mental health) and Physical (PCS) and Mental (MCS) Component Summaries. For the 8 domains, PCS, and MCS, population-normalized *t* scores were calculated (mean [SD], 50 [10]). Scores less than 40 were defined as suboptimal HRQOL; those 40 or more, as optimal HRQOL. Change status in HRQOL was defined as a decline if the status changed from optimal at time 1 to suboptimal at time 2 and as persistently suboptimal if the status was suboptimal at both time 1 and time 2.

#### Predictive Factors for HRQOL

Three distinct types of factors (sociodemographic, lifestyle, and health state) were collected between time 0 and time 1 and used in a prediction model of future suboptimal HRQOL at time 2 and a decline in HRQOL between time 1 and time 2. Sociodemographic predictive factors included age at survey completion, sex, race and ethnicity, educational attainment, employment status, annual household income, marital status, living arrangement, insurance coverage, and primary care or oncology visit. Lifestyle predictive factors included cigarette smoking, physical activity, and body weight per recommendations by the US Centers for Disease Control and Prevention.

Predictive factors for health state included CHCs, emotional distress, and neurocognitive functional status. These factors were selected as potential predictive factors as opposed to cancer treatment, given our assumption that adverse health states were derived from cancer treatment, and adverse health states have direct effects on HRQOL impairment.^20,21^ Moreover, cancer treatments may be less modifiable for future interventions compared with CHCs, emotional distress, and neurocognitive functional status.

Consistent with previous CCSS studies,^22^ 137 individual CHCs were graded using the modified Common Terminology Criteria for Adverse Events, version 4.03, and identified as present if the grade was 2 (moderate), 3 (severe or disabling), or 4 (life-threatening). Organ-specific CHCs were classified as present if any corresponding conditions within an organ group was present. The 11 individual CHC organ groups (visual, hearing, speech, respiratory, cardiovascular, gastrointestinal tract, renal, musculoskeletal, neurological, hematologic, and endocrinological) were included in analyses.

Emotional distress experienced during the prior 7 days was measured using the Brief Symptom Inventory 18,^23^ which included anxiety, depression, and somatization. Age- and sex-adjusted *t* scores 63 or greater above the normative mean were classified as impaired.

Neurocognitive problems experienced during the past 2 months were measured using the CCSS–Neurocognitive Questionnaire,^24^ which included validated scales for emotional regulation, organization, task efficiency, and memory. The emotional regulation and organization domains assessed executive function, whereas task efficiency and memory domains addressed attention, processing speed, and both working and long-term memory. For each domain, age- and sex-adjusted *t* scores 1 SD or more above the normative mean were classified as impaired.

### Statistical Analyses

Data were analyzed from June 19, 2019, to February 2, 2022. To address study aims 1 and 2, PCS and MCS were used as the primary outcomes of interest and the 8 HRQOL domains as secondary outcomes. Using all survivors for this study, multivariable logistic regression analysis identified risk factors at time 1 for suboptimal PCS and MCS (reference category consisted of optimal PCS and MCS) at time 2, adjusting for age, sex, race and ethnicity, and PCS and MCS at time 1. Restricted to participants with optimal HRQOL at time 1, multivariable logistic regression analysis identified risk factors at time 0 and time 1 for a decline in PCS and MCS (reference category, persistently optimal PCS and MCS) between time 1 and time 2 using backward variable selection (stopping at *P* < .05), adjusting for age at time 0, change between time 0 and time 1, and sex, race and ethnicity, and years between time 1 and time 2. For each model, the sample was randomly split into a training data set (80%) for model development and a test data set (20%) for validation. The area under the receiver operating characteristic curve was used to evaluate prediction performance.

To address aim 3, predicted probabilities of all survivors were calculated to generate 3 risk groups (high, medium, and low) for predicting suboptimal PCS and MCS, and 2 risk groups (high and low) for predicting a decline in PCS and MCS. The risk groupings were designed to identify high-risk individuals to have 40% or higher probabilities for future suboptimal and declining PCS and MCS and to distinguish them from individuals at medium and low risk. Two-sided *P* < .05 indicated statistical significance. All statistical analyses were perfomed using SAS, version 9.4 (SAS Institute Inc).

## Results

### Participant Characteristics

Of 4755 survivors, 2623 (55.2%) were women and 2132 (44.8%) were men. A total of 1686 survivors (35.5%) were treated for solid tumors; 1631 (34.3%), for leukemia; 994 (20.9%), for lymphoma; and 444 (9.3%), for malignant neoplasms of the central nervous system (Table 1). The mean (SD) attained ages were 24.3 (7.6) years at time 0, 32.3 (7.4) years at time 1, and 43.8 (7.4) years at time 2. The mean (SD) years from diagnosis were 15.8 (4.7) to time 0, 23.6 (4.5) to time 1, and 35.1 (4.5) to time 2.

**Table 1.** Characteristics of the Study Participants^a^

Characteristic	Assessment		
	Time 0^b^	Time 1	Time 2
**Demographic**			
Age, y			
Mean (SD)	24.5 (7.6)	32.2 (7.4)^c^	43.8 (7.4)^c^
Median (range)	24.3 (8.5-45.9)	32.0 (18.0-53.7)^c^	43.6 (28.4-65.9)^c^
Time since cancer diagnosis, y			
Mean (SD)	15.8 (4.7)	23.6 (4.5)^c^	35.1 (4.5)^c^
Median (range)	15.3 (6.4-29.4)	23.0 (16.1-34.3)^c^	34.6 (27.6-46.4)^c^
Sex			
Male	2132 (44.8)	NA	NA
Female	2623 (55.2)	NA	NA
Race and ethnicity			
Hispanic	164 (3.5)	NA	NA
Non-Hispanic Black	123 (2.6)	NA	NA
Non-Hispanic White	4333 (91.5)	NA	NA
Other^d^	118 (2.5)	NA	NA
Educational attainment			
Did not complete high school	1254 (27.3)	105 (2.2)^c^	59 (1.2)^c^
High school or GED	571 (12.4)	488 (10.3)	353 (7.4)
Some college	1303 (28.3)	1644 (34.6)	1190 (25.0)
College graduate or postgraduate	1473 (32.0)	2517 (52.9)	3153 (66.3)
Employment status			
Employed			
Full-time	NA	3178 (67.5)	3156 (66.9)
Part-time	NA	616 (13.1)	475 (10.1)
Unemployed	NA	917 (19.5)	1086 (23.0)
Annual household income, $			
<20 000	700 (14.7)	422 (8.9)	408 (8.6)
20 000-39 999	2488 (52.3)	910 (19.1)	1788 (37.6)
40 000-59 999		873 (18.3)	
60 000-79 999	1484 (31.2)	771 (16.2)	
≥80 000		1255 (26.4)	2208 (46.4)
Unknown	83 (1.7)	524 (11.0)	351 (7.4)
No. of household members supporting this income			
1	NA	962 (20.6)	959 (20.4)
2	NA	1278 (27.3)	1284 (27.4)
3	NA	947 (20.2)	909 (19.4)
4	NA	950 (20.3)	1013 (21.6)
5	NA	372 (8.0)	348 (7.4)
6	NA	129 (2.8)	118 (2.5)
7	NA	26 (0.5)	41 (0.9)
8	NA	7 (0.1)	8 (0.2)
≥9	NA	6 (0.1)	10 (0.2)
Marital status			
Married or living with partner	1585 (34.0)	2463 (52.3)^c^	3169 (67.0)^c^
Widowed, divorced, or separated	195 (4.2)	344 (7.3)	593 (12.5)
Single	2881 (61.8)	1904 (40.4)	971 (20.5)
Living arrangement			
Living independently	NA	3842 (80.8)	4281 (90.0)
Living dependently	NA	913 (19.2)	474 (10.0)
Health insurance coverage			
Insured or Canadian residence	4209 (89.5)	4250 (89.9)	4459 (94.2)^c^
Uninsured	493 (10.5)	479 (10.1)	274 (5.8)
Primary care or oncology visits^e^			
Yes (≥1 visit)	2127 (44.7)	1484 (31.2)^c^	1491 (31.3)^c^
No	2628 (55.3)	3271 (68.8)	3264 (68.6)
Cigarette smoking			
Never	3813 (80.5)	3398 (72.7)^c^	2842 (63.2)^c^
Past	403 (8.5)	675 (14.4)	1239 (27.6)
Current	522 (11.0)	601 (12.9)	413 (9.2)
Physical activity^f^			
Active	1277 (27.8)	3006 (63.9)^c^	2734 (58.4)^c^
Inactive	3310 (72.2)	1695 (36.1)	1944 (41.5)
Body weight status			
Underweight or normal weight	3065 (66.3)	2412 (51.8)^c^	1794 (38.3)^c^
Overweight	1079 (23.3)	1384 (29.7)	1559 (33.3)
Obese	478 (10.3)	858 (18.4)	1330 (28.4)
**Grades 2-4 CHCs^g^**			
Visual or eye disorders			
Yes	370 (7.8)	417 (8.8)	561 (11.8)^c^
No	4385 (92.2)	4338 (91.2)	4194 (88.2)
Hearing disorders			
Yes	175 (3.7)	228 (4.8)^h^	331 (7.0)^c^
No	4580 (96.3)	4527 (95.2)	4424 (93.0)
Speech disorders			
Yes	7 (0.1)	8 (0.2)	10 (0.2)
No	4748 (99.9)	4747 (99.8)	4745 (99.8)
Respiratory disorders			
Yes	295 (6.2)	374 (7.9)^h^	466 (9.8)^c^
No	4460 (93.8)	4381 (92.1)	4289 (90.2)
Cardiovascular disorders			
Yes	595 (12.5)	1122 (23.6)^c^	2038 (42.9)^c^
No	4160 (87.5)	3633 (76.4)	2717 (57.1)
Gastrointestinal tract disorders			
Yes	445 (9.3)	583 (12.3)^c^	760 (16.0)^c^
No	4310 (90.6)	4172 (87.7)	3995 (84.0)
Renal disorders			
Yes	39 (0.8)	49 (1.0)	78 (1.6)^c^
No	4716 (99.2)	4706 (99.0)	4677 (98.3)
Musculoskeletal disorders			
Yes	335 (7.0)	358 (7.5)	402 (8.5)^i^
No	4420 (92.9)	4397 (92.5)	4353 (91.5)
Neurological disorders			
Yes	574 (12.1)	654 (13.7)^i^	810 (17.0)^c^
No	4181 (87.9)	4101 (86.2)	3945 (83.0)
Hematologic disorders			
Yes^j^	1 (0.02)	4 (0.1)	5 (0.1)
No	4754 (100)	4751 (99.9)	4750 (99.9)
Endocrinological disorders			
Yes	1005 (21.1)	1322 (27.8)^c^	1910 (40.2)^c^
No	3750 (78.9)	3433 (72.2)	2845 (59.8)
**Emotional distress**			
Anxiety			
Impaired	201 (5.7)	290 (6.7)	242 (5.1)
Not impaired	3300 (94.3)	4031 (93.3)	4493 (94.9)
Depression			
Impaired	298 (8.5)	443 (10.2)^h^	371 (7.8)
Not impaired	3204 (91.5)	3879 (89.7)	4363 (92.2)
Somatization			
Impaired	227 (6.5)	499 (11.5)^c^	388 (8.2)^h^
Not impaired	3274 (93.5)	3821 (88.4)	4346 (91.8)
Global Severity Index			
Impaired	223 (6.4)	370 (8.6)^c^	300 (6.3)
Not impaired	3276 (93.6)	3949 (91.4)	4433 (93.7)
**Neurocognitive function**			
Memory			
Impaired	NA	526 (12.6)	1140 (24.1)
Not impaired	NA	3659 (87.4)	3581 (75.9)
Task efficiency			
Impaired	NA	834 (19.9)	1004 (21.3)
Not impaired	NA	3349 (80.1)	3716 (78.7)
Organization			
Impaired	NA	496 (11.9)	454 (9.6)
Not impaired	NA	3689 (88.1)	4263 (90.4)
Emotional regulation			
Impaired	NA	489 (11.7)	544 (11.5)
Not impaired	NA	3695 (88.3)	4178 (88.5)
Cancer diagnosis			
Leukemia	1631 (34.3)	NA	NA
Central nervous system tumor	444 (9.3)	NA	NA
Hodgkin lymphoma	627 (13.2)	NA	NA
Non–Hodgkin lymphoma	367 (7.7)	NA	NA
Wilms tumor	472 (9.9)	NA	NA
Neuroblastoma	321 (6.7)	NA	NA
Sarcoma	439 (9.2)	NA	NA
Bone tumor	454 (9.5)	NA	NA
**Chemotherapy**			
Methotrexate			
Yes	1985 (45.2)	NA	NA
No	2407 (54.8)	NA	NA
Corticosteroid			
Yes	1899 (45.1)	NA	NA
No	2313 (54.9)	NA	NA
Anthracyclines			
Yes	1745 (39.1)	NA	NA
No	2712 (60.8)	NA	NA
Alkylating agents			
Yes	2206 (49.6)	NA	NA
No	2241 (50.4)	NA	NA
Other chemotherapy			
Yes	323 (7.3)	NA	NA
No	4080 (92.7)	NA	NA
**Radiation therapy**			
Brain			
Yes	1369 (31.0)	NA	NA
No	3052 (69.0)	NA	NA
Chest			
Yes	1130 (25.6)	NA	NA
No	3290 (74.4)	NA	NA
Abdominal			
Yes	1082 (24.5)	NA	NA
No	3339 (75.5)	NA	NA
Pelvic			
Yes	841 (19.0)	NA	NA
No	3580 (81.0)	NA	NA
Other			
Yes	226 (5.1)	NA	NA
No	4196 (94.9)	NA	NA
**Surgery**			
Splenectomy			
Yes	428 (9.6)	NA	NA
No	4035 (90.4)	NA	NA
Nephrectomy			
Yes	440 (9.9)	NA	NA
No	4023 (90.1)	NA	NA
Amputation			
Yes	228 (5.1)	NA	NA
No	4235 (94.9)	NA	NA
Other major surgery			
Yes	2359 (52.9)	NA	NA
No	2104 (47.1)	NA	NA
Relapse of malignant neoplasms before assessment time			
Yes	202 (4.2)	323 (6.8)^c^	604 (12.7)^c^
No	4553 (95.7)	4432 (93.2)	4151 (87.3)
Time since previous assessment, y			
Mean (SD)	NA	7.7 (1.2)	11.6 (0.7)
Median (range)	NA	8.0 (0.5-11.5)	11.5 (9.4-13.8)

Abbreviations: CCSS, Childhood Cancer Survivor Study; CHC, chronic health condition; GED, General Educational Development diploma; HRQOL, health-related quality of life; NA, not applicable.

^a^ The total sample size includes 4755 CCSS survivors of childhood cancer who participated in surveys at times 0, 1, and 2. Unless indicated otherwise, data are expressed as No. (%) of patients. Owing to missing data, numbers for some characteristics do not total 4755. Percentages have been rounded and may not total 100.

^b^ The enrollment criteria for the CCSS’s baseline (time 0) include 5-year survival from cancer diagnosis and younger than 21 years at the time of diagnosis of cancer. Some survivors were younger than 18 years at time 0.

^c^ Comparisons between time 1 and time 0 and between time 2 and time 0: *P* < .001.

^d^ Includes American Indian or Alaska Native, Asian or Pacific Islander, or unknown race or ethnicity.

^e^ Captures the concept of physician visits related to previous cancer or similar illness in the past 2 years. Survivors who had more physician visits may have more severe health conditions or concerns about health conditions that may be associated with future suboptimal HRQOL or a decline in HRQOL.

^f^ Based on available physical activity items, active status at time 0 was defined as performing sport or exercise that causes sweating or breathing hard for at least 20 minutes for at least 4 days in a week, whereas active status at time 1 was defined as performing at least 150 minutes of moderate aerobic physical activity or 75 minutes of vigorous physical activity in a week.

^g^ We used modified Common Terminology Criteria for Adverse Events, version 4.03, grades 2 to 4 CHCs (moderate, severe, and life-threatening, respectively) instead of grades 3 and 4 CHCs (severe and life-threatening, respectively) to classify the risk of suboptimal HRQOL or a decline in HRQOL. This approach will allow clinicians to identify survivors who have moderate severity of CHCs (grade 2) to provide early interventions for preventing progression to severe or life-threatening CHCs (grades 3 and 4).

^h^ Comparisons between time 1 and time 0 and between time 2 and time 0: *P* < .01.

^i^ Comparisons between time 1 and time 0 and between time 2 and time 0: *P* < .05.

^j^ Fisher exact test was used for cell with fewer than 5 survivors.

### Prevalence of Suboptimal PCS and MCS and Change Over Time

For PCS, 371 of 3337 survivors (11.1%) had suboptimal status at time 1 and 548 of 3753 (14.6%) at time 2; 285 of 3294 (8.7%) had a decline in PCS from time 1 to time 2; and 176 of 3294 (5.3%) had persistently suboptimal PCS at both time 1 and time 2 (eTable 3 in the Supplement). For MCS, 583 of 3337 (17.5%) had suboptimal status at time 1; 575 of 3753 (15.3%) had suboptimal status at time 2; 278 of 3294 (8.4%) had a decline in MCS from time 1 to time 2, and 206 of 3294 (6.3%) had persistently suboptimal MCS at both time 1 and time 2.

### Risk Factors for Future Suboptimal PCS and MCS

Multivariable models identified distinct sociodemographic, lifestyle, and health state variables at time 1 as risk factors for future suboptimal PCS HRQOL (vs future optimal PCS as the reference category) at time 2 (Table 2). Risk factors included prevalent respiratory (odds ratio [OR], 1.69 [95% CI, 1.17-2.45]), cardiovascular (OR, 1.68 [95% CI, 1.31-2.16]), gastrointestinal tract (OR, 1.64 [95% CI, 1.21-2.24]), musculoskeletal (OR, 2.29 [95% CI, 1.57-3.33]), neurological (OR, 2.33 [95% CI, 1.73-3.14]), and endocrine (OR, 1.54 [95% CI, 1.19-1.98]) CHCs of grades 2 to 4, as well as having depression (OR, 1.63 [95% CI, 1.16-2.30]) and memory deficits (OR, 1.55 [95% CI, 1.14-2.12]).

**Table 2.** Predictive Factors Associated With Suboptimal PCS and MCS at Time 2 and the Performance of Prediction Models

Predictive factor	Suboptimal PCS at time 2^a^		Suboptimal MCS at time 2^a^	
	OR (95% CI)	*P* value	OR (95% CI)	*P* value
Age at time 1, y (continuous)	1.04 (1.02-1.06)	<.001	1.00 (0.98-1.01)	.82
Sex				
Female	1.62 (1.26-2.09)	<.001	1.26 (1.00-1.58)	.05
Male	[Reference]	NA	[Reference]	NA
Race and ethnicity				
Hispanic	0.96 (0.49-1.91)	.92	1.03 (0.55-1.94)	.92
Non-Hispanic Black	1.74 (0.85-3.55)	.13	0.69 (0.29-1.66)	.41
Non-Hispanic White	[Reference]	NA	[Reference]	NA
Other^b^	0.92 (0.42-1.99)	.83	0.64 (0.30-1.36)	.25
Marital status at time 1				
Widowed, divorced, or separated	NA	NA	1.46 (1.00-2.15)	.05
Single	NA	NA	1.04 (0.80-1.35)	.79
Married or living with partner	NA	NA	[Reference]	NA
Educational attainment at time 1				
Less than high school	2.08 (1.09-3.98)	.03	NA	NA
High school or GED	1.66 (1.15-2.42)	.007	NA	NA
Some college	1.19 (0.91-1.54)	.20	NA	NA
College graduate or postgraduate	[Reference]	NA	NA	NA
Employment status at time 1				
Part-time	1.01 (0.72-1.43)	.95	1.04 (0.76-1.43)	.82
Unemployed	1.38 (1.03-1.85)	.03	1.27 (0.97-1.67)	.08
Full-time	[Reference]	NA	[Reference]	NA
Annual household income at time 1, $				
<20 000	1.34 (0.89-2.03)	.16	NA	NA
20 000-39 999	1.43 (1.04-1.97)	.03	NA	NA
40 000-59 999	0.99 (0.71-1.39)	.96	NA	NA
60 000-79 999	1.23 (0.88-1.73)	.22	NA	NA
≥80 000	[Reference]	NA	NA	NA
Primary care or oncology visits at time 1^c^				
No	0.94 (0.73-1.20)	.62	0.78 (0.62-0.97)	.03
Yes (≥1 visit)	[Reference]	NA	[Reference]	NA
Cigarette smoking at time 1				
Past	NA	NA	1.15 (0.84-1.57)	.38
Current	NA	NA	1.42 (1.05-1.93)	.02
Never	NA	NA	[Reference]	NA
Physical activity at time 1^d^				
Inactive	1.49 (1.19-1.88)	<.001	NA	NA
Active	[Reference]	NA	NA	NA
Respiratory disorders at time 1^e^				
Yes	1.69 (1.17-2.45)	.005	NA	NA
No	[Reference]	NA	NA	NA
Cardiovascular disorders at time 1^e^				
Yes	1.68 (1.31-2.16)	<.001	NA	NA
No	[Reference]	NA	NA	NA
Gastrointestinal tract disorders at time 1^e^				
Yes	1.64 (1.21-2.24)	.002	NA	NA
No	[Reference]	NA	NA	NA
Musculoskeletal disorders at time 1^e^				
Yes	2.29 (1.57-3.33)	<.001	NA	NA
No	[Reference]	NA	NA	NA
Neurological disorders at time 1^e^				
Yes	2.33 (1.73-3.14)	<.001	1.58 (1.19-2.10)	.002
No	[Reference]	NA	[Reference]	NA
Endocrinological disorders at time 1^e^				
Yes	1.54 (1.19-1.98)	<.001	NA	NA
No	[Reference]	NA	NA	NA
Depression at time 1				
Yes	1.63 (1.16-2.30)	.005	2.26 (1.63-3.12)	<.001
No	[Reference]	NA	[Reference]	NA
Somatization at time 1				
Yes	NA	NA	1.48 (1.10-1.99)	.01
No	NA	NA	[Reference]	NA
Memory at time 1				
Yes	1.55 (1.14-2.12)	.006	NA	NA
No	[Reference]	NA	NA	NA
Task efficiency at time 1				
Yes	NA	NA	1.94 (1.52-2.48)	<.001
No	NA	NA	[Reference]	NA
HRQOL at time 1				
Suboptimal	4.26 (3.22-5.64)	<.001	2.48 (1.90-3.25)	<.001
Optimal	[Reference]	NA	[Reference]	NA
Model performance (AUROC)				
Training data set	0.80 (0.78-0.83)	NA	0.75 (0.73-0.78)	NA
Test data set (95% CI)	0.82 (0.77-0.86)	NA	0.74 (0.69-0.79)	NA

Abbreviations: AUROC, area under the receiver operating characteristic curve; GED, General Educational Development diploma; HRQOL, health-related quality of life; MCS, Mental Component Summary; NA, not applicable; PCS, Physical Component Summary.

^a^ Independent variables were added to the model based on backward selection with cutoff *P* < .05. The backward selection includes 3 steps for selecting significant social variables, lifestyle variables, and health variables. In each step, the previous selected variables were forced in the model of the next step. For the models of suboptimal outcome at time 2, sex, race and ethnicity, age at time 1, and time 1 HRQOL were forced in all models. Reference category consists of optimal PCS or MCS at time 2.

^b^ Includes American Indian or Alaska Native, Asian or Pacific Islander, or unknown race or ethnicity.

^c^ Captures the concept of physician visits related to previous cancer or similar illness in the past 2 years. Survivors who had more physician visits may have more severe health conditions or concerns about health conditions that may be associated with future suboptimal HRQOL or a decline in HRQOL.

^d^ Active status at time 0 was defined as performing sport or exercise that causes sweating or breathing hard for at least 20 minutes for at least 4 days in a week, whereas active status at time 1 was defined as performing at least 150 minutes of moderate aerobic physical activity or 75 minutes of vigorous physical activity in a week.

^e^ We used modified Common Terminology Criteria for Adverse Events, version 4.03, grades 2 to 4 chronic health conditions (CHCs; moderate, severe, and life-threatening, respectively) instead of grades 3 and 4 CHCs (severe and life-threatening, respectively) to classify the risk of suboptimal HRQOL or a decline in HRQOL. This approach will allow clinicians to identify survivors who have moderate severity of CHCs (grade 2) to provide early interventions for preventing progression to severe or life-threatening CHCs (grades 3 and 4).

Higher risk of future suboptimal MCS (vs future optimal MCS as the reference category) at time 2 was associated with being current vs never cigarette smokers (OR, 1.42 [95% CI, 1.05-1.93]), having a neurological CHC of grades 2 to 4 (OR, 1.58 [95% CI, 1.19-2.10]), and having depression (OR, 2.26 [95% CI, 1.63-3.12]), somatization (OR, 1.48 [95% CI, 1.10-1.99]), and impaired task efficiency (OR, 1.94 [95% CI, 1.52-2.48]). Similar patterns of risk factors were observed for suboptimal status for 8 HRQOL domains (eTable 4 in the Supplement).

### Risk Factors for Decline in PCS and MCS Over Time

Multivariable models identified sociodemographic, lifestyle, and health state variables at time 0 or time 1 as risk factors for a decline in HRQOL between time 1 and time 2 (Table 3). Most of the risk factors for a decline in PCS (vs persistently optimal PCS between time 1 and time 2 as the reference category) were identified at time 1, consisting of having an annual household income less than $20 000 vs $80 000 or more (OR, 2.00 [95% CI, 1.21-3.30]); being physically inactive vs active (OR, 1.63 [95% CI, 1.25-2.13]); experiencing various CHCs of grades 2 to 4 based on individual organ systems, including cardiovascular (OR, 1.53 [95% CI, 1.14-2.06]), gastrointestinal tract (OR, 1.89 [95% CI, 1.32-2.69]), neurological (OR, 2.16 [95% CI, 1.51-3.10]), respiratory (OR, 1.66 [95% CI, 1.06-2.59]), and endocrine (OR, 2.25 [95% CI, 1.44-3.52]) systems; and having depression (OR, 1.79 [95% CI, 1.20-2.67]) and impaired memory (OR, 1.58 [95% CI, 1.08-2.30]). In addition, being female (OR, 1.67 [95% CI, 1.25-2.24]), having obesity vs underweight or normal weight at time 0 (OR, 1.97 [95% CI, 1.32-2.92]), and the presence of musculoskeletal CHCs at time 0 (OR, 2.24 [95% CI, 1.39-3.61]) were associated with a decline in PCS.

**Table 3.** Predictive Factors Associated With a Decline in PCS and MCS From Time 1 to Time 2 and the Performance of Prediction Models

Predictive factor	Decline from optimal to suboptimal from time 1 to time 2^a^			
	In PCS		In MCS	
	OR (95% CI)	*P* value	OR (95% CI)	*P* value
Age, y				
Time 1	1.04 (1.02-1.06)	<.001	1.00 (0.98-1.03)	.83
From time 0 to time 1	1.00 (0.82-1.23)	.99	0.87 (0.71-1.08)	.21
Sex				
Female	1.67 (1.25-2.24)	<.001	1.21 (0.89-1.64)	.22
Male	[Reference]	NA	[Reference]	NA
Race/ethnicity				
Hispanic	0.87 (0.36-2.10)	.76	1.19 (0.56-2.54)	.65
Non-Hispanic Black	1.07 (0.42-2.73)	.88	0.63 (0.21-1.90)	.41
Non-Hispanic White	[Reference]	NA	[Reference]	NA
Other^b^	1.00 (0.43-2.31)	>.99	0.45 (0.14-1.48)	.19
Educational attainment				
Time 1				
Less than high school	1.87 (0.87-4.02)	.11	NA	NA
High school or GED	1.59 (1.02-2.46)	.04	NA	NA
Some college	1.13 (0.83-1.53)	.43	NA	NA
College graduate or postgraduate	[Reference]	NA	NA	NA
From time 0 to time 1				
Improved	NA	NA	1.33 (0.94-1.89)	.11
No changes	NA	NA	[Reference]	NA
Employment status, time 1				
Part-time	0.96 (0.64-1.44)	.85	1.29 (0.86-1.94)	.23
Unemployed	1.59 (1.13-2.26)	.008	1.68 (1.19-2.38)	.003
Full-time	[Reference]	NA	[Reference]	NA
Annual household income, time 1, $				
<20 000	2.00 (1.21-3.30)	.007	NA	NA
20 000-39 999	1.36 (0.90-2.07)	.14	NA	NA
40 000-59 999	1.14 (0.78-1.68)	.50	NA	NA
60 000-79 999	1.32 (0.87-2.00)	.20	NA	NA
≥80 000	[Reference]	NA	NA	NA
Living arrangement, time 1				
Living dependently	NA	NA	1.16 (0.81-1.67)	.41
Living independently	NA	NA	[Reference]	NA
Primary care or oncology visits^c^				
Time 1				
No	NA	NA	0.70 (0.52-0.94)	.02
Yes (≥1 visit)	NA	NA	[Reference]	NA
Time 0				
No	0.89 (0.68-1.18)	.43	NA	NA
Yes (≥1 visit)	[Reference]	NA	NA	NA
Cigarette smoking, time 1				
Past	NA	NA	1.19 (0.79-1.80)	.40
Current	NA	NA	2.03 (1.37-3.00)	<.001
Never	NA	NA	[Reference]	NA
Physical activity^d^				
Time 0				
Inactive	NA	NA	1.48 (1.05-2.09)	.03
Active	NA	NA	[Reference]	NA
Time 1				
Inactive	1.63 (1.25-2.13)	<.001	1.30 (0.97-1.73)	.08
Active	[Reference]	NA	[Reference]	NA
Body weight status, time 0				
Underweight or normal weight	[Reference]	NA	NA	NA
Overweight	1.35 (0.98-1.86)	.06	NA	NA
Obese	1.97 (1.32-2.92)	<.001	NA	NA
Respiratory disorders, time 1^e^				
Yes	1.66 (1.06-2.59)	.03	NA	NA
No	[Reference]	NA	NA	NA
Cardiovascular disorders, time 1^e^				
Yes	1.53 (1.14-2.06)	.005	NA	NA
No	[Reference]	NA	NA	NA
Gastrointestinal disorders, time 1^e^				
Yes	1.89 (1.32-2.69)	<.001	NA	NA
No	[Reference]	NA	NA	NA
Musculoskeletal disorders, time 0^e^				
Yes	2.24 (1.39-3.61)	<.001	NA	NA
No	[Reference]	NA	NA	NA
Neurological disorders, time 1^e^				
Yes	2.16 (1.51-3.10)	<.001	NA	NA
No	[Reference]	NA	NA	NA
Endocrinological disorders^e^				
Time 1				
Yes	2.25 (1.44-3.52)	<.001	NA	NA
No	[Reference]	NA	NA	NA
Time 0				
Yes	0.59 (0.36-0.96)	.03	NA	NA
No	[Reference]	NA	NA	NA
Depression, time 1				
Yes	1.79 (1.20-2.67)	.004	4.29 (2.44-7.55)	<.001
No	[Reference]	NA	[Reference]	NA
Somatization, time 1				
Yes	NA	NA	1.63 (1.05-2.53)	.03
No	NA	NA	[Reference]	NA
Memory, time 1				
Yes	1.58 (1.08-2.30)	.02	NA	NA
No	[Reference]	NA	NA	NA
Task efficiency, time 1				
Yes	NA	NA	1.90 (1.34-2.68)	<.001
No	NA	NA	[Reference]	NA
Organization, time 1				
Yes	NA	NA	1.67 (1.12-2.48)	.01
No	NA	NA	[Reference]	NA
Model performance (AUROC)				
Training data set	0.75 (0.71-0.78)	NA	0.72 (0.68-0.75)	NA
Test data set (95% CI)	0.74 (0.67-0.81)	NA	0.68 (0.60-0.75)	NA

Abbreviations: AUROC, area under the receiver operating characteristic curve; GED, General Educational Development diploma; MCS, Mental Component Summary; NA, not applicable; PCS, Physical Component Summary.

^a^ Independent variables were added to the model based on backward selection with cutoff *P* < .05. The backward selection includes 3 steps for selecting significant social variables, lifestyle variables, and health variables. In each step, the previous selected variables were forced in the model of the next step. For the models of HRQOL changes from time 1 to time 2, sex, race and ethnicity, age at time 1, and years between time 1 and time 2 were forced in all models. Reference category consists of optimal PCS or MCS from time 1 to time 2.

^b^ Includes American Indian or Alaska Native, Asian or Pacific Islander, or unknown.

^c^ Captures the concept of physician visits related to previous cancer or similar illness in the past 2 years. Survivors who had more physician visits may have more severe health conditions or concerns about health conditions that may be associated with future suboptimal HRQOL or a decline in HRQOL.

^d^ Active status at time 0 was defined as performing sport or exercise that causes sweating or breathing hard for at least 20 minutes for at least 4 days in a week, whereas active status at time 1 was defined as performing at least 150 minutes of moderate aerobic physical activity or 75 minutes of vigorous physical activity in a week.

^e^ We used modified Common Terminology Criteria for Adverse Events, version 4.03, grades 2 to 4 chronic health conditions (CHCs; moderate, severe, and life-threatening, respectively) instead of grades 3 and 4 CHCs (severe and life-threatening, respectively) to classify the risk of suboptimal HRQOL or a decline in HRQOL. This approach will allow clinicians to identify survivors who have moderate severity of CHCs (grade 2) to provide early interventions for preventing progression to severe or life-threatening CHCs (grades 3 and 4).

Higher risk for a decline in MCS (vs persistently optimal MCS between time 1 and time 2 as the reference category) was associated with being a current vs never cigarette smoker (OR, 2.03 [95% CI, 1.37-3.00]), being physically inactive vs active at time 0 (OR, 1.48 [95% CI, 1.05-2.09]), being unemployed vs having full-time employment (OR, 1.68; [95% CI, 1.19-2.38]), and having depression (OR, 4.29 [95% CI, 2.44-7.55]), somatization (OR, 1.63 [95% CI, 1.05-2.53]), impaired task efficiency (OR, 1.90 [95% CI, 1.34-2.68]), and impaired organization (OR, 1.67 [95% CI, 1.12-2.48]) at time 1. Similar patterns of risk factors were found in 8 HRQOL domains (eTable 5 in the Supplement).

### Performance of Prediction Models

Risk prediction models performed satisfactorily for predicting suboptimal and declining PCS and MCS (Tables 2 and 3). For the prediction of suboptimal HRQOL, the areas under the receiver operating characteristic curve were higher for PCS compared with MCS models (0.82 [95% CI, 0.77-0.86] for PCS vs 0.74 [95% CI, 0.69-0.79) for MCS with test data sets). For a decline in HRQOL prediction, the areas under the receiver operating characteristics curve were higher for PCS compared with MCS models (0.74 [95% CI, 0.67-0.81] for PCS vs 0.68 [95% CI, 0.60-0.75] for MCS with test data sets).

**Figure 1.**
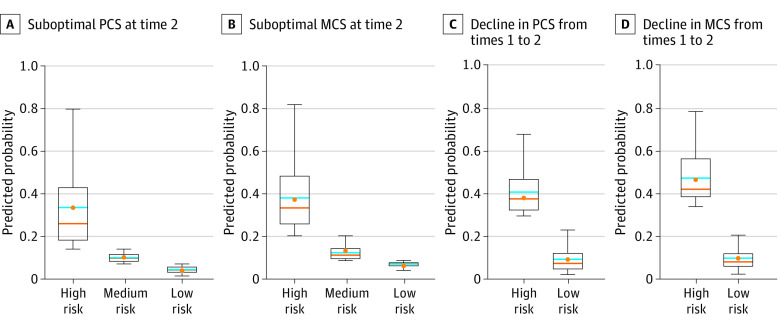
Predicted Probabilities by Predicted Risk Group of Suboptimal and Declining Health-Related Quality of Life In each box and whisker plot, the top whisker indicates the maximum predicted probability; the bottom whisker indicates the minimum predicted probability. Blue lines indicate the mean of the predicted probability; orange lines, the median of the predicted probability; and orange dots, the observed probability. MCS indicates Mental Component Summary; PCS, Physical Component Summary.

**Figure 2.**
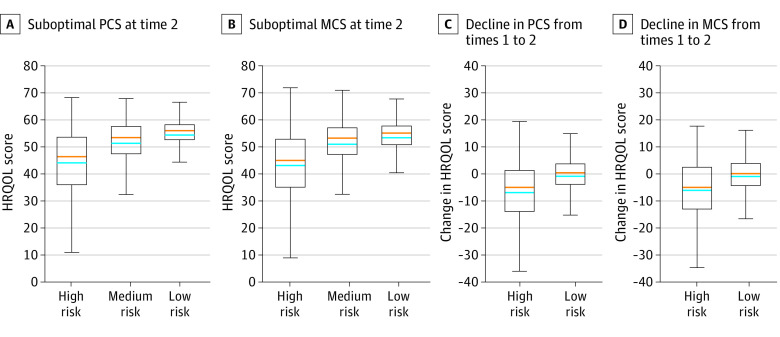
Observed Health-Related Quality of Life (HRQOL) Physical Component Summary (PCS) and Mental Component Summary (MCS) Scores and Their Changes by Predicted Risk Group In each box and whisker plot, the top whisker indicates the maximum suboptimal HRQOL score (A and B) and maximum change of HRQOL score (C and D); the bottom whisker indicates the minimum suboptimal HRQOL score (A and B) and minimum change of HRQOL score (C and D). Blue lines indicate the mean of suboptimal HRQOL scores (A and B) and the change of HRQOL scores (C and D); orange lines indicate the median of suboptimal HRQOL scores (A and B) and the change of HRQOL scores (C and D).

Figure 1 shows the predicted probabilities of future suboptimal and declining PCS and MCS for individuals by their risk groups based on the risk factors listed in Tables 2 and 3. Survivors from the high-risk HRQOL group had 40% or higher predicted probabilities of future suboptimal and declining PCS and MCS. The predicted probabilities of future suboptimal HRQOL among survivors from the high-risk group were appreciably higher than among survivors from the medium- or low-risk groups (Figure 1A and B); and the predicted probabilities of a decline in HRQOL for survivors from the high-risk group were appreciably higher than those for survivors from the low-risk group (Figure 1C and D). Furthermore, for survivors in the high-risk group, the scores of PCS and MCS were approximately 40 points (ie, 1 SD) below the norm of 50 (Figure 2A and B) and the decrease from time 1 to time 2 in PCS and MCS scores were above 5 points (Figure 2C and D), which met the threshold of minimally important difference.^25^

## Discussion

We developed and validated risk prediction models to identify a population of survivors of childhood cancer who are at higher risk of suboptimal and declining physical and mental HRQOL. With a 40% probability, we were successful in identifying the most vulnerable survivors based on sociodemographic factors, health behaviors, presence of grade 2 to 4 CHCs, and impaired neurocognitive and psychological status. Despite an abundant literature on HRQOL among survivors of childhood cancer, the present study fills a crucial gap by extending our ability to predict survivors who will experience poor HRQOL or a decline in HRQOL from optimal to suboptimal after many years of survival. This information is needed to ensure surveillance of these risk factors is conducted within the context of follow-up care appointments. When the presence of these factors is identified, recommendations for referral to appropriate interventions are warranted. Furthermore, this information can help guide the design of future interventions to prevent HRQOL decline based on the modifiable factors identified.

We found that 5.3% to 6.3% of survivors reported persistently suboptimal HRQOL and 8.4% to 8.7% of survivors reported a decline in HRQOL from time 1 to time 2 during a mean of 11.6 years. With more than 500 000 survivors of childhood cancer in US alone, this finding translates to a substantial number of survivors who are currently living with suboptimal HRQOL decades after their cancer diagnosis and treatment. The proportion of survivors demonstrating a decline from optimal to suboptimal HRQOL differs from that reported among healthy individuals.^26,27,28^

Not surprisingly, moderate to severe or life-threatening CHCs were found to predict physical HRQOL decline. Given that nearly 100% of cancer survivors develop a CHC by 50 years of age,^6^ there is an urgent need to minimize the effects of subsequent CHCs on HRQOL. Ideally, survivors of childhood cancer should receive lifelong, cancer-specific follow-up care,^29,30,31^ including the ongoing surveillance and prevention of recurrent and new cancers and of other CHCs.^32^ However, less than 50% of survivors of childhood cancer attend long-term follow-up clinics,^33,34^ and survivors demonstrate poor knowledge of their unique risks for treatment-related late effects, particularly among young adults.^35^ Access to follow-up care might be complicated by a number of factors, including socioeconomic status,^36^ which in the present study was shown to be associated with decreased HRQOL. Thus, innovative and accessible interventions to engage survivors in follow-up care are needed.

Current smoking, physical inactivity, and poor mental health were found to be associated with decreased mental HRQOL, which are modifiable risk factors for intervention. Evidence-based interventions targeting smoking cessation,^37^ physical inactivity,^38^ and emotional distress^39^ already exist for childhood cancer survivors and the general population. In the absence of this, clinicians should familiarize themselves with local resources to facilitate referral of survivors to receive appropriate support services. Nevertheless, the evidence from the present study provides a foundation for the design and testing of future clinical trials to target these risk factors, with the ultimate goal of improving HRQOL among survivors of childhood cancer.

### Limitations

This study has some limitations. First, there were some differences in those survivors who completed the time 1 and time 2 questionnaires compared with those who completed the time 1 questionnaire only. Those who completed both questionnaires were more likely to be women and non-Hispanic White; to have a college graduate or postgraduate educational level; and to have a higher annual household income, which is consistent with research conducted in community samples.^40^ Our findings, therefore, may not be generalizable to larger, more diverse survivor cohorts, and further exploration for the role of social determinants of health in predicting HRQOL among a more diverse sample of survivors is needed. Importantly, we did not include environmental factors (eg, neighborhood adversity) that have been shown to be associated with poor HRQOL. Cancer survivors living in disadvantaged social and built environments (eg, food deserts, neighborhoods lacking parks and recreational facilities) likely experience poorer HRQOL.^41^ Improving HRQOL in cancer survivors, especially those living in disadvantaged neighborhoods, provides a promising avenue for future exploration.^42^ Finally, this study develops and validates a risk prediction model for HRQOL based on the CCSS cohort alone. We did not validate our risk prediction model with an external non-CCSS survivorship cohort because comparable cohorts that include the same risk factors at time 0 and time 1, and the same time intervals between time 0 and time 1 and between time 1 and time 2 are currently not available. Future comparable survivorship cohorts, once available, are warranted to validate our risk prediction model.

## Conclusions

To our knowledge, this prognostic study is the first and largest study to examine prediction models by evaluating how risk factors at one point in time predict HRQOL at a later point. Our findings support the existing literature exploring factors associated with HRQOL among survivors of childhood cancer and enable the identification of at-risk survivors who may benefit from preventive interventions. Clinicians should screen for these factors during follow-up appointments and refer patients for interventions when appropriate. We found that CHCs could predict a decline in physical HRQOL, whereas cigarette smoking, physical inactivity, and emotional-neurocognitive impairments could predict a decline in mental HRQOL. The implications of this work allow clinicians to speak to cancer survivors about the likelihood of maintaining poor or worsening HRQOL in the presence of specific risk factors or, more importantly, how alterations or elimination of risk factors might help improve HRQOL over time. Given the growing number of cancer survivors, there continues to be an urgent need to identify those survivors who are at greatest risk and implement novel research toward improving their HRQOL.

## Notes

Supplement. **eFigure.** Flowchart for Including Eligible CCSS Cancer Survivors in This Study **eTable 1.** Comparisons Between CCSS Cancer Survivors Who Participated in Time 1 Only (Excluded From This Study) and Survivors Who Participated in Both Times 1 and 2 (Included in This Study) **eTable 2.** HRQOL at Time 1 Among CCSS Cancer Survivors Who Participated in Time 1 Only (Excluded From This Study) and in Both Times 1 and 2 (Included in This Study) **eTable 3.** HRQOL at Times 1 and 2 and the Change Status From Times 1 to 2 Among CCSS Cancer Survivors Included in This Study (n = 3804) **eTable 4.** Predictive Factors for Suboptimal HRQOL at Time 2 by 8 Domains of SF-36 and the Performance of Prediction Models **eTable 5.** Predictive Factors for a Decline in HRQOL From Times 1 to 2 by 8 Domains of SF-36 and the Performance of Prediction Models
