# Supplementary material for: Development and Validation of Models to Predict Poor Health-Related Quality of Life Among Adult Survivors of Childhood Cancer
Source: JAMA Netw Open. 2022 Aug 17;5(8):e2227225. doi: 10.1001/jamanetworkopen.2022.27225 (PMC9386537; doi:10.1001/jamanetworkopen.2022.27225)
Supplement: Supplement. — eFigure. Flowchart for Including Eligible CCSS Cancer Survivors in This Study eTable 1. Comparisons Between CCSS Cancer Survivors Who Participated in Time 1 Only (Excluded From This Study) and Survivors Who Participated in Both Times 1 and 2 (Included in This Study) eTable 2. HRQOL at Time 1 Among CCSS Cancer Survivors Who Participated in Time 1 Only (Excluded From This Study) and in Both Times 1 and 2 (Included in This Study) eTable 3. HRQOL at Times 1 and 2 and the Change Status From Times 1 to 2 Among CCSS Cancer Survivors Included in This Study (n = 3804) eTable 4. Predictive Factors for Suboptimal HRQOL at Time 2 by 8 Domains of SF-36 and the Performance of Prediction Models eTable 5. Predictive Factors for a Decline in HRQOL From Times 1 to 2 by 8 Domains of SF-36 and the Performance of Prediction Models [file jamanetwopen-e2227225-s001.pdf]

# Supplementary Online Content

Schulte F, Chen Y, Yasui Y, et al. Development and validation of models to predict poor health-related quality of life among adult survivors of childhood cancer. *JAMA Netw Open*. 2022;5(8):e2227225. doi:10.1001/jamanetworkopen.2022.27225

**eFigure.** Flowchart for Including Eligible CCSS Cancer Survivors in This Study
**eTable 1.** Comparisons Between CCSS Cancer Survivors Who Participated in Time 1 Only (Excluded From This Study) and Survivors Who Participated in Both Times 1 and 2 (Included in This Study)
**eTable 2.** HRQOL at Time 1 Among CCSS Cancer Survivors Who Participated in Time 1 Only (Excluded From This Study) and in Both Times 1 and 2 (Included in This Study)
**eTable 3.** HRQOL at Times 1 and 2 and the Change Status From Times 1 to 2 Among CCSS Cancer Survivors Included in This Study (n = 3804)
**eTable 4.** Predictive Factors for Suboptimal HRQOL at Time 2 by 8 Domains of SF-36 and the Performance of Prediction Models
**eTable 5.** Predictive Factors for a Decline in HRQOL From Times 1 to 2 by 8 Domains of SF-36 and the Performance of Prediction Models

This supplementary material has been provided by the authors to give readers additional information about their work.

© 2022 Schulte F et al. *JAMA Network Open*.

eFigure. Flowchart for Including Eligible CCSS Cancer Survivors in This Study

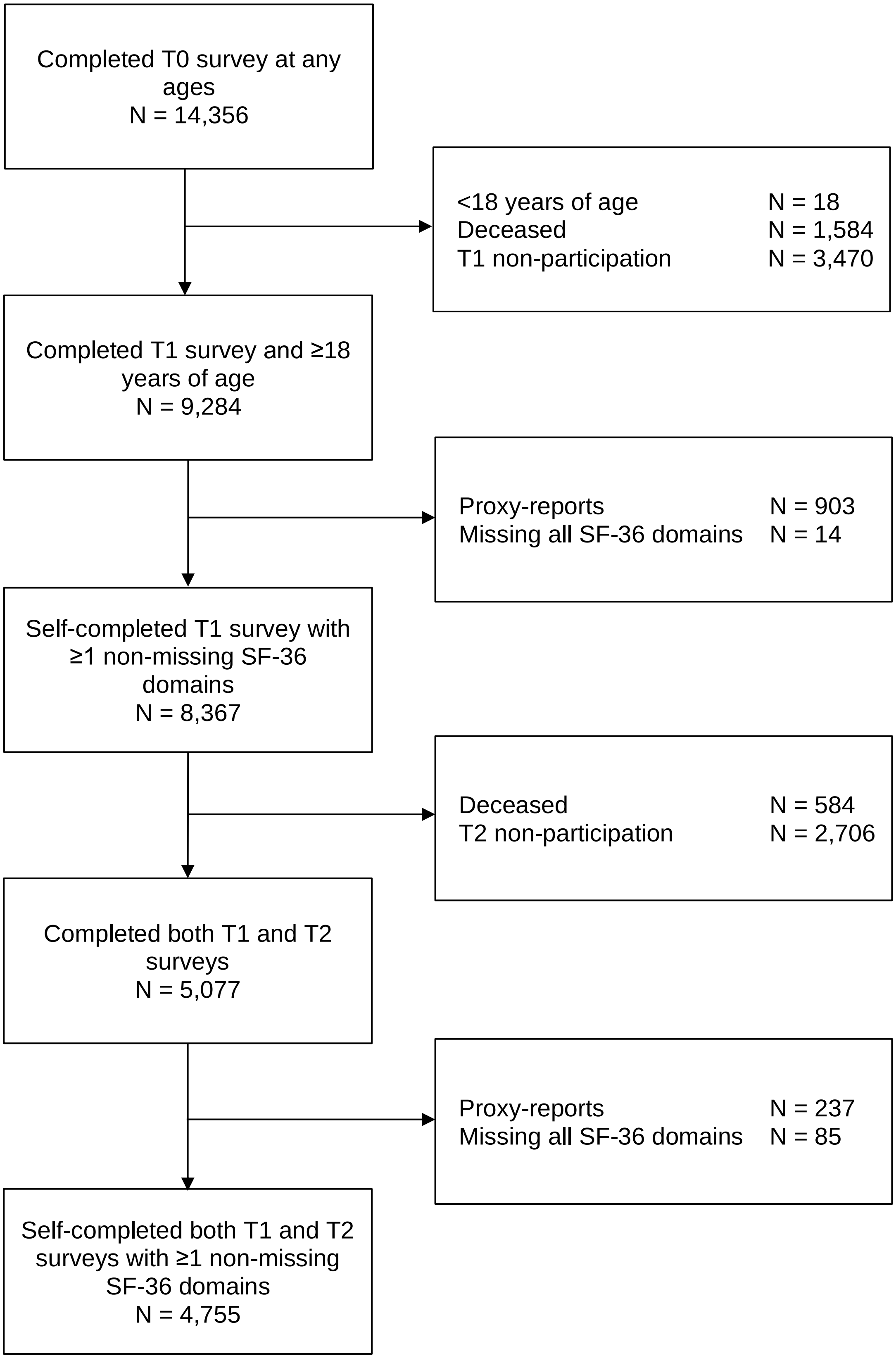


© 2022 Schulte F et al. *JAMA Network Open*.

eTable 1. Comparisons Between CCSS Cancer Survivors Who Participated in Time 1 Only (Excluded From This Study) and Survivors Who Participated in Both Times 1 and 2 (Included in This Study)

| **Characteristics of study sample** | | **Survivors completed T1 survey only (N = 3,612)** | **Survivors completed both T1 and T2 surveys and used in this study (N = 4,755)†** | **t- or $X^2$-statistic (P-value)** |
|---|---|---|---|---|
| | | **Mean (SD/range) or N (%)** | **Mean (SD/range) or N (%)** | |
| Age in years at T1 | Mean (SD) | 32.0 (7.6) | 32.2 (7.4) | 0.32 |
| | Median (range) | 31.5 (25.8, 37.4) | 32.0 (18.0, 53.7) | 0.12 |
| Years since cancer diagnosis | Mean (SD) | 23.8 (4.6) | 23.6 (4.5) | 0.01 |
| | Median (range) | 23.2 (19.8, 27.5) | 23.0 (16.1, 34.3) | 0.02 |
| Sex | Male | 1990 (55.1) | 2132 (44.8) | <0.001 |
| | Female | 1622 (44.9) | 2623 (55.2) | |
| Race/ethnicity | White, non-Hispanic | 3123 (86.9) | 4333 (91.5) | <0.001 |
| | Black, non-Hispanic | 158 (4.4) | 123 (2.6) | |
| | Hispanic | 199 (5.5) | 164 (3.5) | |
| | Other§ | 115 (3.2) | 118 (2.5) | |
| Educational attainment at T1 | Did not complete high school | 200 (5.5) | 105 (2.2) | <0.001 |
| | High school/GED | 676 (18.7) | 488 (10.3) | |
| | Some college | 1464 (40.5) | 1644 (34.6) | |
| | College graduate or postgraduate | 1272 (35.2) | 2517 (52.9) | |
| Employment status at T1 | Working full-time | 2260 (63.2) | 3178 (67.5) | <0.001 |
| | Working part-time | 414 (11.6) | 616 (13.1) | |
| | Unemployed | 900 (25.2) | 917 (19.5) | |
| Annual household income at T1 (US dollars) | <$20,000 | 470 (13.0) | 422 (8.9) | <0.001 |
| | $20,000 - $39,999 | 773 (21.4) | 910 (19.1) | |
| | $40,000 - $59,999 | 613 (17.0) | 873 (18.4) | |
| | $60,000 - $79,999 | 432 (12.0) | 771 (16.2) | |
| | ≥$80,000 | 688 (19.0) | 1255 (26.4) | |
| | Unknown | 636 (17.6) | 524 (11.0) | |
| | 1 | 736 (21.0) | 962 (20.6) | 0.10 |
| | 2 | 915 (26.1) | 1278 (27.3) | |

© 2022 Schulte F et al. *JAMA Network Open*.

| | | | | | |
|---|---|---|---|---|---|
| # of household members supporting this income | | 3 | 788 (22.5) | 947 (20.2) | |
| | | 4 | 665 (19.0) | 950 (20.3) | |
| | | 5 | 282 (8.0) | 372 (8.0) | |
| | | 6 | 82 (2.3) | 129 (2.8) | |
| | | 7 | 19 (0.5) | 26 (0.6) | |
| | | 8 | 13 (0.4) | 7 (0.1) | |
| | | 9 or more | 4 (0.1) | 6 (0.1) | |
| Marital status at T1 | | Married/Living with partner | 1628 (45.4) | 2463 (52.3) | <0.001 |
| | | Widowed/Divorced/Separated | 304 (8.5) | 344 (7.3) | |
| | | Single | 1655 (46.1) | 1904 (40.4) | |
| Living arrangement at T1 | | Living independently | 2598 (71.9) | 3842 (80.8) | <0.001 |
| | | Living dependently | 1014 (28.1) | 913 (19.2) | |
| Health insurance coverage at T1 | | Insured or Canadian residence | 3053 (85.3) | 4250 (89.9) | <0.001 |
| | | Uninsured | 527 (14.7) | 479 (10.1) | |
| Primary care or oncology visits at T1 | | Yes (≥1 visits) | 1060 (29.3) | 1484 (31.2) | 0.07 |
| | | No | 2552 (70.7) | 3271 (68.8) | |
| Cigarette smoking at T1 | | Never smoker | 2248 (63.9) | 3398 (72.7) | <0.001 |
| | | Past smoker | 529 (15.0) | 675 (14.4) | |
| | | Current smoker | 741 (21.1) | 601 (12.9) | |
| Physical activity at T1 | | Active | 2088 (58.9) | 3006 (63.9) | <0.001 |
| | | Inactive | 1459 (41.1) | 1695 (36.1) | |
| Body weight status at T1 | | Underweight/normal weight | 1647 (46.9) | 2412 (51.8) | <0.001 |
| | | Overweight | 1068 (30.4) | 1384 (29.7) | |
| | | Obese | 799 (22.7) | 858 (18.4) | |
| Chronic health conditions at T1 | Vision/eye disorders | Yes | 363 (10.0) | 417 (8.8) | 0.5 |
| | | No | 3249 (90.0) | 4338 (91.2) | |
| | Hearing disorders | Yes | 173 (4.8) | 228 (4.8) | 0.99 |
| | | No | 3439 (95.2) | 4527 (95.2) | |
| | Speech disorders | Yes | 6 (0.2) | 8 (0.2) | 0.98 |
| | | No | 3606 (99.8) | 4747 (99.8) | |
| | Respiratory disorders | Yes | 202 (5.6) | 374 (7.9) | <0.001 |
| | | No | 3410 (94.4) | 4381 (92.1) | |
| | Cardiovascular disorders | Yes | 799 (22.1) | 1122 (23.6) | 0.11 |
| | | No | 2813 (77.9) | 3633 (76.4) | |
| | Gastrointestinal disorders | Yes | 291 (8.1) | 583 (12.3) | <0.001 |
| | | No | 3321 (91.9) | 4172 (87.7) | |
| | Renal disorders | Yes | 40 (1.1) | 49 (1.0) | 0.73 |

© 2022 Schulte F et al. *JAMA Network Open*.

| | | | | | |
|---|---|---|---|---|---|
| | | No | 3572 (98.9) | 4706 (99.0) | |
| | Musculoskeletal disorders | Yes | 210 (5.8) | 358 (7.5) | 0.002 |
| | | No | 3402 (94.2) | 4397 (92.5) | |
| | Neurologic disorders | Yes | 468 (13.0) | 654 (13.8) | 0.29 |
| | | No | 3144 (87.0) | 4101 (86.3) | |
| | Hematologic disorders‡ | Yes | 3 (0.1) | 4 (0.1) | 1.00 |
| | | No | 3609 (99.9) | 4751 (99.9) | |
| | Endocrinological disorders | Yes | 817 (22.6) | 1322 (27.8) | <0.001 |
| | | No | 2795 (77.4) | 3433 (72.2) | |
| Emotional distress at T1 | Anxiety | Impaired | 261 (9.2) | 290 (6.7) | <0.001 |
| | | Not impaired | 2578 (90.8) | 4031 (93.3) | |
| | Depression | Impaired | 397 (14.0) | 443 (10.2) | <0.001 |
| | | Not impaired | 2441 (86.0) | 3879 (89.8) | |
| | Somatization | Impaired | 503 (17.7) | 499 (11.6) | <0.001 |
| | | Not impaired | 2335 (82.3) | 3821 (88.5) | |
| | Global severity index | Impaired | 359 (12.7) | 370 (8.6) | <0.001 |
| | | Not impaired | 2478 (87.3) | 3949 (91.5) | |
| Neurocognitive function at T1 | Memory | Impaired | 428 (16.2) | 526 (12.6) | <0.001 |
| | | Not impaired | 2218 (83.8) | 3659 (87.5) | |
| | Task efficiency | Impaired | 707 (26.7) | 834 (19.9) | <0.001 |
| | | Not impaired | 1937 (73.3) | 3349 (80.1) | |
| | Organization | Impaired | 361 (13.6) | 496 (11.9) | 0.03 |
| | | Not impaired | 2287 (86.4) | 3689 (88.2) | |
| | Emotional regulation | Impaired | 371 (14.0) | 489 (11.7) | 0.005 |
| | | Not impaired | 2274 (86.0) | 3695 (88.3) | |
| Cancer diagnosis | Leukemia | | 1226 (33.9) | 1631 (34.3) | <0.001 |
| | Central nervous system tumor | | 438 (12.1) | 444 (9.3) | |
| | Hodgkin's lymphoma | | 511 (14.1) | 627 (13.2) | |
| | Non-Hodgkin's lymphoma | | 283 (7.8) | 367 (7.7) | |
| | Wilms' tumor | | 318 (8.8) | 472 (9.9) | |
| | Neuroblastoma | | 229 (6.3) | 321 (6.8) | |
| | Sarcoma | | 323 (8.9) | 439 (9.2) | |
| | Bone tumor | | 284 (7.9) | 454 (9.5) | |
| Chemotherapy | Methotrexate | Yes | 1364 (43.7) | 1985 (45.2) | 0.20 |
| | | No | 1758 (56.3) | 2407 (54.8) | |
| | Corticosteroid | Yes | 1359 (45.8) | 1899 (45.1) | 0.56 |
| | | No | 1609 (54.2) | 2313 (54.9) | |

© 2022 Schulte F et al. *JAMA Network Open*.

| | | | | | |
|---|---|---|---|---|---|
| | Anthracyclines | Yes | 1227 (38.6) | 1745 (39.2) | 0.65 |
| | | No | 1949 (61.4) | 2712 (60.8) | |
| | Alkylating agents | Yes | 1590 (50.2) | 2206 (49.6) | 0.64 |
| | | No | 1580 (49.8) | 2241 (50.4) | |
| | Other chemotherapy | Yes | 191 (6.1) | 323 (7.3) | 0.03 |
| | | No | 2952 (93.9) | 4080 (92.7) | |
| Radiation therapy | Brain irradiation | Yes | 1097 (34.9) | 1369 (31.0) | <0.001 |
| | | No | 2046 (65.1) | 3052 (69.0) | |
| | Chest irradiation | Yes | 889 (28.3) | 1130 (25.6) | 0.008 |
| | | No | 2252 (71.7) | 3290 (74.4) | |
| | Abdominal irradiation | Yes | 867 (27.6) | 1082 (24.5) | 0.002 |
| | | No | 2274 (72.4) | 3339 (75.5) | |
| | Pelvic irradiation | Yes | 695 (22.1) | 841 (19.0) | <0.001 |
| | | No | 2446 (77.9) | 3580 (81.0) | |
| | Other radiation therapy | Yes | 148 (4.7) | 226 (5.1) | 0.42 |
| | | No | 2997 (95.3) | 4196 (94.9) | |
| Surgery | Splenectomy | Yes | 347 (10.9) | 428 (9.6) | 0.06 |
| | | No | 2834 (89.1) | 4035 (90.4) | |
| | Nephrectomy | Yes | 272 (8.6) | 440 (9.9) | 0.05 |
| | | No | 2909 (91.4) | 4023 (90.1) | |
| | Amputation | Yes | 133 (4.2) | 228 (5.1) | 0.06 |
| | | No | 3048 (95.8) | 4235 (94.9) | |
| | Other major surgery | Yes | 1748 (55.0) | 2359 (52.9) | 0.07 |
| | | No | 1433 (45.0) | 2104 (47.1) | |
| Relapse/second malignant neoplasms | | Yes | 347 (9.6) | 323 (6.8) | <0.001 |
| | | No | 3265 (90.4) | 4432 (93.2) | |
| Years from cancer diagnosis to T1 | | Mean (SD) | 23.8 (4.6) | 23.6 (4.5) | 0.01 |
| | | Median (range) | 23.2 (19.8, 27.5) | 23.0 (19.7, 27.0) | 0.02 |

GED = General educational development diploma

† Sample size of this study is 4,755 CCSS cancer survivors

‡ Fisher's exact test was used for cell with <5 survivors

§ Other: American Indian / Alaska Native, Asian or Pacific Islander, or unknown

© 2022 Schulte F et al. *JAMA Network Open*.

eTable 2. HRQOL at Time 1 Among CCSS Cancer Survivors Who Participated in Time 1 Only (Excluded From This Study) and in Both Times 1 and 2 (Included in This Study)

| Specific HRQOL domain | Survivors at T1 only (excluded from this study) | | Survivors in both T1 and T2 (included in this study) | | P-value |
|---|---|---|---|---|---|
| | Suboptimal HRQOL | Optimal HRQOL | Suboptimal HRQOL | Optimal HRQOL | |
| | N (%) | N (%) | N (%) | N (%) | |
| PF at T1 | 509 (14.1) | 3098 (85.9) | 443 (9.3) | 4303 (90.7) | <0.001 |
| RP at T1 | 607 (16.9) | 2989 (83.1) | 562 (11.9) | 4171 (88.1) | <0.001 |
| BP at T1 | 751 (20.8) | 2854 (79.2) | 827 (17.4) | 3914 (82.6) | <0.001 |
| GH at T1 | 754 (28.1) | 1926 (71.9) | 875 (20.8) | 3337 (79.2) | <0.001 |
| VT at T1 | 741 (27.6) | 1944 (72.4) | 987 (23.4) | 3236 (76.6) | <0.001 |
| SF at T1 | 607 (16.8) | 3000 (83.2) | 559 (11.8) | 4191 (88.2) | <0.001 |
| RE at T1 | 702 (19.5) | 2889 (80.5) | 789 (16.7) | 3938 (83.3) | <0.001 |
| MH at T1 | 594 (22.1) | 2091 (77.9) | 704 (16.7) | 3518 (83.3) | <0.001 |
| PCS at T1 | 494 (18.6) | 2161 (81.4) | 483 (11.6) | 3692 (88.4) | <0.001 |
| MCS at T1 | 545 (20.5) | 2110 (79.5) | 732 (17.5) | 3443 (82.5) | 0.002 |

PF: physical functioning, RP: role limitation due to physical health problems, BP: bodily pain, GH: general health perceptions, VT: vitality, SF: social functioning, RE: role limitation due to emotional health problems, MH: mental health; PCS = Physical Component Summary; MCS = Mental Component Summary

© 2022 Schulte F et al. *JAMA Network Open*.

eTable 3. HRQOL at Times 1 and 2 and the Change Status From Times 1 to 2 Among CCSS Cancer Survivors Included in This Study (n = 3804)†

| **Specific HRQOL domain** | **T1** | | **T2** | | **Change from T1 to T2** | | | |
|---|---|---|---|---|---|---|---|---|
| | **Suboptimal** | **Optimal** | **Suboptimal** | **Optimal** | **Declining** | **Persistently suboptimal** | **Improved** | **Persistently optimal** |
| | N (%) | N (%) | N (%) | N (%) | N (%) | N (%) | N (%) | N (%) |
| PF | 344 (9.1) | 3454 (90.9) | 446 (11.8) | 3347 (88.2) | 273 (7.2) | 172 (4.5) | 171 (4.5) | 3171 (83.7) |
| RP | 449 (11.9) | 3337 (88.1) | 594 (15.7) | 3195 (84.3) | 403 (10.7) | 186 (4.9) | 260 (6.9) | 2922 (77.5) |
| BP | 654 (17.2) | 3140 (82.8) | 506 (13.3) | 3291 (86.7) | 268 (7.1) | 236 (6.2) | 418 (11.0) | 2865 (75.7) |
| GH | 669 (19.9) | 2696 (80.1) | 860 (22.8) | 2918 (77.2) | 361 (10.8) | 390 (11.7) | 275 (8.2) | 2317 (69.3) |
| VT | 789 (23.4) | 2586 (76.6) | 672 (17.7) | 3115 (82.3) | 289 (8.6) | 296 (8.8) | 490 (14.6) | 2286 (68.0) |
| SF | 435 (11.4) | 3366 (88.6) | 501 (13.2) | 3293 (86.8) | 343 (9.0) | 158 (4.2) | 277 (7.3) | 3013 (79.5) |
| RE | 635 (16.8) | 3147 (83.2) | 445 (11.7) | 3343 (88.3) | 281 (7.5) | 158 (4.2) | 473 (12.6) | 2854 (75.8) |
| MH | 565 (16.7) | 2810 (83.3) | 513 (13.5) | 3276 (86.5) | 237 (7.0) | 200 (5.9) | 363 (10.8) | 2563 (76.2) |
| PCS | 371 (11.1) | 2966 (88.9) | 548 (14.6) | 3205 (85.4) | 285 (8.7) | 176 (5.3) | 191 (5.8) | 2642 (80.2) |
| MCS | 583 (17.5) | 2754 (82.5) | 575 (15.3) | 3178 (84.7) | 278 (8.4) | 206 (6.3) | 362 (11.0) | 2448 (74.3) |

PF: physical functioning, RP: role limitation due to physical health problems, BP: bodily pain, GH: general health perceptions, VT: vitality, SF: social functioning, RE: role limitation due to emotional health problems, MH: mental health; PCS = Physical Component Summary; MCS = Mental Component Summary

† Based on the training dataset (i.e., 80% of the random sample selected from the entire study sample, N=4,755).

© 2022 Schulte F et al. *JAMA Network Open*.

eTable 4. Predictive Factors for Suboptimal HRQOL at Time 2 by 8 Domains of SF-36 and the Performance of Prediction Models

| Predictor | Level | Suboptimal PF at T2† OR (95%CI; P-value) | Suboptimal RP at T2† OR (95%CI; P-value) | Suboptimal BP at T2† OR (95%CI; P-value) | Suboptimal GH at T2† OR (95%CI; P-value) |
|---|---|---|---|---|---|
| Age in years at T1 | | 1.05 (1.03 - 1.07; <.001) | 1.05 (1.03 - 1.07; <.001) | 1.03 (1.01 - 1.05; <.001) | 1.00 (0.99 - 1.02; 0.53) |
| Sex | Female | 1.73 (1.30 - 2.29; <.001) | 1.36 (1.07 - 1.74; 0.01) | 1.61 (1.26 - 2.07; <.001) | 1.19 (0.97 - 1.46; 0.10) |
| | Male | Reference | Reference | Reference | Reference |
| Race/ethnicity | Black, non-Hispanic | 2.28 (1.15 - 4.52; 0.02) | 1.94 (0.97 - 3.90; 0.06) | 1.45 (0.72 - 2.92; 0.30) | 1.05 (0.53 - 2.10; 0.89) |
| | Hispanic | 0.77 (0.36 - 1.63; 0.49) | 0.96 (0.50 - 1.84; 0.91) | 1.58 (0.88 - 2.84; 0.13) | 1.08 (0.62 - 1.89; 0.79) |
| | Other[§] | 0.59 (0.23 - 1.47; 0.26) | 1.24 (0.62 - 2.50; 0.54) | 0.84 (0.38 - 1.85; 0.66) | 1.12 (0.61 - 2.06; 0.71) |
| | White, non-Hispanic | Reference | Reference | Reference | Reference |
| Marital status at T1 | Widowed/divorced/separated | | | | |
| | Single | | | | |
| | Married/living with partner | | | | |
| Educational attainment at T1 | Less than high school | 2.49 (1.25 - 4.98; 0.01) | 2.03 (1.09 - 3.79; 0.03) | 2.76 (1.51 - 5.05; <.001) | |
| | High school/GED | 1.58 (1.04 - 2.41; 0.03) | 1.62 (1.12 - 2.35; 0.01) | 1.69 (1.17 - 2.44; 0.005) | |
| | Some college | 1.59 (1.20 - 2.11; 0.001) | 1.31 (1.02 - 1.68; 0.03) | 1.40 (1.08 - 1.81; 0.01) | |
| | College graduate or postgraduate | Reference | Reference | Reference | |
| Employment status at T1 | Part-time | 1.06 (0.73 - 1.55; 0.75) | 1.02 (0.73 - 1.43; 0.90) | 0.98 (0.69 - 1.38; 0.89) | 0.91 (0.68 - 1.22; 0.52) |
| | Unemployed | 1.42 (1.03 - 1.95; 0.03) | 1.62 (1.22 - 2.15; <.001) | 1.28 (0.96 - 1.70; 0.09) | 1.26 (0.98 - 1.62; 0.08) |
| | Full-time | | | | |
| Annual household income at T1 | <$20,000 | 1.34 (0.87 - 2.05; 0.19) | 1.28 (0.86 - 1.90; 0.22) | 1.21 (0.81 - 1.82; 0.35) | 1.42 (1.00 - 2.01; 0.05) |
| | $20,000 - $39,999 | 1.15 (0.80 - 1.63; 0.45) | 1.43 (1.06 - 1.94; 0.02) | 1.43 (1.04 - 1.95; 0.3) | 1.31 (1.00 - 1.71; 0.05) |
| | $40,000 - $59,999 | 0.93 (0.64 - 1.34; 0.69) | 0.96 (0.69 - 1.33; 0.80) | 1.11 (0.79 - 1.55; 0.55) | 0.97 (0.74 - 1.29; 0.86) |
| | $60,000 - $79,999 | 0.92 (0.63 - 1.35; 0.67) | 0.90 (0.64 - 1.27; 0.55) | 1.09 (0.77 - 1.54; 0.64) | 1.05 (0.79 - 1.40; 0.73) |
| | ≥$80,000 | Reference | Reference | Reference | Reference |
| Living arrangement at T1 | Living dependently | 1.14 (0.81 - 1.61; 0.46) | 0.98 (0.72 - 1.35; 0.92) | | 1.05 (0.81 - 1.38; 0.70) |
| | Living independently | Reference | Reference | | Reference |
| Primary care or oncology visits at T1 | No | 0.88 (0.68 - 1.15; 0.37) | 0.79 (0.63 - 1.00; 0.05) | | 0.80 (0.65 - 0.98; 0.03) |
| | Yes (≥1 visits) | Reference | Reference | | Reference |
| Cigarette smoking at T1 | Past smoker | | | 1.06 (0.77 - 1.46; 0.73) | |
| | Current smoker | | | 1.21 (0.87 - 1.69; 0.26) | |
| | Never smoker | | | Reference | |
| Physical activity at T1 | Inactive | 1.40 (1.09 - 1.80; 0.009) | 1.29 (1.03 - 1.62; 0.03) | 1.21 (0.96 - 1.53; 0.11) | |
| | Active | Reference | Reference | Reference | |
| Body weight status at T1 | Underweight/normal weight | | Reference | | Reference |
| | Overweight | | 1.12 (0.86 - 1.45; 0.41) | | 1.14 (0.91 - 1.43; 0.26) |
| | Obese | | 1.18 (0.88 - 1.58; 0.26) | | 1.41 (1.10 - 1.81; 0.007) |
| Vision/eye disorders at T1 | Yes | | 1.56 (1.10 - 2.20; 0.01) | | |
| | No | | Reference | | |
| Hearing disorders at T1 | Yes | 1.70 (1.07 - 2.69; 0.02) | | | |
| | No | Reference | | | |
| Respiratory disorders at T1 | Yes | 1.93 (1.31 - 2.86; <.001) | 2.02 (1.43 - 2.86; <.001) | 1.69 (1.18 - 2.43; 0.004) | 2.01 (1.46 - 2.76; <.001) |
| | No | Reference | Reference | Reference | Reference |
| Cardiovascular disorders | Yes | 1.61 (1.23 - 2.12; <.001) | 1.59 (1.24 - 2.02; <.001) | 1.53 (1.19 - 1.96; <.001) | 1.59 (1.27 - 1.97; <.001) |
| | No | Reference | Reference | Reference | Reference |
| Gastrointestinal disorders at T1 | Yes | | | | 1.53 (1.17 - 2.00; 0.002) |
| | No | | | | Reference |
| | Yes | 2.61 (1.76 - 3.88; <.001) | 1.60 (1.09 - 2.37; 0.02) | | |

© 2022 Schulte F et al. *JAMA Network Open*.

| | | | | | |
|---|---|---|---|---|---|
| Musculoskeletal disorders | No | Reference | Reference | | |
| Neurologic disorders at T1 | Yes | 2.60 (1.90 - 3.55; <.001) | 2.51 (1.89 - 3.34; <.001) | 1.87 (1.41 - 2.49; <.001) | 2.07 (1.58 - 2.72; <.001) |
| | No | Reference | Reference | Reference | Reference |
| Endocrinological disorders at T1 | Yes | 1.39 (1.05 - 1.84; 0.02) | 1.49 (1.17 - 1.89; 0.001) | | 1.44 (1.16 - 1.78; <.001) |
| | No | Reference | Reference | | Reference |
| Anxiety at T1 | Yes | | | | 1.57 (1.11 - 2.23; 0.011) |
| | No | | | | Reference |
| Depression at T1 | Yes | | 1.65 (1.19 - 2.31; 0.003) | 1.60 (1.15 - 2.24; 0.006) | |
| | No | | Reference | Reference | |
| Somatization at T1 | Yes | | 1.54 (1.12 - 2.11; 0.008) | 1.73 (1.27 - 2.37; <.001) | |
| | No | | Reference | Reference | |
| Memory at T1 | Yes | | 1.91 (1.43 - 2.55; <.001) | | 1.33 (1.01 - 1.76; 0.04) |
| | No | | Reference | | Reference |
| Emotional regulation at T1 | Yes | 1.78 (1.28 - 2.48; <.001) | | | |
| | No | Reference | | | |
| HRQOL at T1 | Suboptimal | 4.93 (3.58 - 6.79; <.001) | 2.10 (1.56 - 2.82; <.001) | 3.48 (2.70 - 4.49; <.001) | 6.33 (5.13 - 7.80; <.001) |
| | Optimal | Reference | Reference | Reference | Reference |
| | | | | | |
| **Model performance (area under the ROC curve)** | | **Training dataset: 0.83 Test dataset: 0.84 (0.79-0.88)** | **Training dataset: 0.79 Test dataset: 0.79 (0.75-0.83)** | **Training dataset: 0.78 Test dataset: 0.77 (0.72-0.82)** | **Training dataset: 0.79 Test dataset: 0.77 (0.73-0.82)** |

© 2022 Schulte F et al. *JAMA Network Open*.

eTable 4. (Con't)

| Predictor | Level | Suboptimal VT at T2† OR (95%CI; P-value) | Suboptimal SF at T2† OR (95%CI; P-value) | Suboptimal RE at T2† OR (95%CI; P-value) | Suboptimal MH at T2† OR (95%CI; P-value) |
|---|---|---|---|---|---|
| Age in years at T1 | | 1.00 (0.99 - 1.02; 0.60) | 1.01 (0.99 - 1.03; 0.17) | 1.02 (1.01 - 1.04; 0.009) | 0.99 (0.97 - 1.00; 0.11) |
| Sex | Female | 1.74 (1.39 - 2.18; <.001) | 1.29 (1.02 - 1.64; 0.04) | 1.44 (1.11 - 1.88; 0.006) | 1.25 (0.98 - 1.58; 0.07) |
| | Male | Reference | Reference | Reference | Reference |
| Race/ethnicity | Black, non-Hispanic | 1.19 (0.58 - 2.42; 0.63) | 0.77 (0.34 - 1.76; 0.54) | 1.82 (0.90 - 3.69; 0.09) | 1.22 (0.57 - 2.62; 0.60) |
| | Hispanic | 0.97 (0.53 - 1.75; 0.91) | 1.77 (1.00 - 3.12; 0.05) | 0.94 (0.46 - 1.91; 0.86) | 1.51 (0.85 - 2.68; 0.16) |
| | Other§ | 1.25 (0.68 - 2.31; 0.48) | 1.07 (0.53 - 2.16; 0.85) | 1.13 (0.55 - 2.34; 0.74) | 0.83 (0.40 - 1.71; 0.61) |
| | White, non-Hispanic | Reference | Reference | Reference | Reference |
| Marital status at T1 | Widowed/divorced/separated | | | | |
| | Single | | | | |
| | Married/living with partner | | | | |
| Educational attainment at T1 | Less than high school | | 1.58 (0.84 - 2.95; 0.15) | 1.44 (0.75 - 2.77; 0.27) | |
| | High school/GED | | 1.22 (0.83 - 1.79; 0.31) | 1.02 (0.66 - 1.56; 0.95) | |
| | Some college | | 1.31 (1.01 - 1.70; 0.04) | 1.36 (1.04 - 1.79; 0.03) | |
| | College graduate or postgraduate | | Reference | Reference | |
| Employment status at T1 | Part-time | 0.67 (0.49 - 0.93; 0.02) | 0.87 (0.61 - 1.23; 0.42) | 1.20 (0.84 - 1.71; 0.32) | 0.66 (0.46 - 0.95; 0.02) |
| | Unemployed | 1.14 (0.88 - 1.48; 0.31) | 1.30 (0.98 - 1.73; 0.07) | 1.81 (1.35 - 2.42; <.001) | 1.18 (0.89 - 1.56; 0.24) |
| | Full-time | Reference | Reference | Reference | Reference |
| Annual household income at T1 | <$20,000 | 1.77 (1.24 - 2.54; 0.002) | 1.27 (0.87 - 1.86; 0.22) | 1.10 (0.74 - 1.64; 0.64) | |
| | $20,000 - $39,999 | 1.46 (1.10 - 1.95; 0.009) | 1.01 (0.73 - 1.38; 0.97) | 0.92 (0.65 - 1.29; 0.63) | |
| | $40,000 - $59,999 | 1.33 (0.99 - 1.79; 0.06) | 0.96 (0.69 - 1.34; 0.82) | 0.95 (0.67 - 1.36; 0.80) | |
| | $60,000 - $79,999 | 1.10 (0.80 - 1.50; 0.57) | 0.80 (0.56 - 1.15; 0.23) | 0.90 (0.62 - 1.30; 0.57) | |
| | ≥$80,000 | Reference | Reference | Reference | |
| Living arrangement | Living dependently | | 1.13 (0.84 - 1.52; 0.43) | 1.10 (0.80 - 1.51; 0.57) | 1.20 (0.90 - 1.60; 0.22) |
| | Living independently | | Reference | Reference | Reference |
| Health insurance at T1 | Uninsured | | | | |
| | Insured/Canadian resident | | | | |
| Primary care or oncology visits at T1 | No | 0.68 (0.55 - 0.85; <.001) | 0.81 (0.64 - 1.03; 0.08) | 0.91 (0.70 - 1.17; 0.45) | 0.85 (0.67 - 1.08; 0.18) |
| | Yes (≥1 visits) | Reference | Reference | Reference | Reference |
| Cigarette smoking at T1 | Past smoker | 1.32 (0.98 - 1.76; 0.07) | 1.01 (0.72 - 1.41; 0.97) | 1.15 (0.82 - 1.63; 0.42) | 1.15 (0.83 - 1.59; 0.41) |
| | Current smoker | 1.32 (0.99 - 1.78; 0.06) | 1.47 (1.07 - 2.02; 0.02) | 1.36 (0.96 - 1.92; 0.08) | 1.61 (1.19 - 2.17; 0.002) |
| | Never smoker | Reference | Reference | Reference | Reference |
| Physical activity at T1 | Inactive | 1.54 (1.25 - 1.90; <.001) | | 1.28 (1.00 - 1.63; 0.050) | |
| | Active | Reference | | Reference | |
| Hearing disorders at T1 | Yes | | 1.61 (1.05 - 2.46; 0.03) | | |
| | No | | Reference | | |
| Cardiovascular disorders at T1 | Yes | 1.35 (1.06 - 1.70; 0.01) | 1.54 (1.20 - 1.98; <.001) | | |
| | No | Reference | Reference | | |
| Gastrointestinal disorders at T1 | Yes | 1.38 (1.03 - 1.84; 0.03) | 1.62 (1.19 - 2.20; 0.002) | | |
| | No | Reference | Reference | | |
| Musculoskeletal disorders at T1 | Yes | | | | 1.66 (1.09 - 2.52; 0.02) |
| | No | | | | Reference |
| Neurologic disorders at T1 | Yes | 1.70 (1.29 - 2.23; <.001) | 1.56 (1.15 - 2.10; 0.004) | 1.88 (1.39 - 2.54; <.001) | 1.52 (1.13 - 2.06; 0.006) |
| | No | Reference | Reference | Reference | Reference |

© 2022 Schulte F et al. *JAMA Network Open*.

| | | | | | |
|---|---|---|---|---|---|
| Endocrinological disorders at T1 | Yes | | | | |
| | No | | | | |
| Anxiety at T1 | Yes | | 1.61 (1.10 - 2.37; 0.02) | | 1.47 (1.01 - 2.15; 0.04) |
| | No | | Reference | | Reference |
| Depression at T1 | Yes | 2.31 (1.71 - 3.13; <.001) | | 2.16 (1.53 - 3.03; <.001) | 1.94 (1.37 - 2.75; <.001) |
| | No | Reference | | Reference | Reference |
| Somatization at T1 | Yes | 1.39 (1.04 - 1.87; 0.03) | 1.57 (1.15 - 2.16; 0.005) | 1.82 (1.32 - 2.49; <.001) | |
| | No | Reference | Reference | Reference | |
| Memory at T1 | Yes | 1.45 (1.10 - 1.93; 0.009) | | | 1.40 (1.03 - 1.91; 0.03) |
| | No | Reference | | | Reference |
| Task efficiency at T1 | Yes | | 1.32 (1.00 - 1.74; 0.05) | 1.97 (1.50 - 2.59; <.001) | 1.52 (1.14 - 2.01; 0.004) |
| | No | | Reference | Reference | Reference |
| Organization at T1 | Yes | | | | |
| | No | | | | |
| Emotional regulation at T1 | Yes | 1.36 (1.01 - 1.82; 0.04) | 1.48 (1.08 - 2.03; 0.02) | | |
| | No | Reference | Reference | | |
| HRQOL at T1 | Suboptimal | 2.65 (2.11 - 3.33; <.001) | 2.26 (1.67 - 3.06; <.001) | 1.60 (1.20 - 2.13; 0.001) | 2.95 (2.21 - 3.94; <.001) |
| | Optimal | Reference | Reference | Reference | Reference |
| | | | | | |
| **Model performance (area under the ROC curve)** | | **Training dataset: 0.77 Test dataset: 0.72 (0.67-0.77)** | **Training dataset: 0.73 Test dataset: 0.73 (0.67-0.77)** | **Training dataset: 0.76 Test dataset: 0.75 (0.69-0.81)** | **Training dataset: 0.75 Test dataset: 0.70 (0.65-0.76)** |

PF: physical functioning, RP: role limitation due to physical health problems, BP: bodily pain, GH: general health perceptions, VT: vitality, SF: social functioning, RE: role limitation due to emotional health problems, MH: mental health; GED = General educational development diploma; ROC = Receiver Operating Characteristic

Based on backward selection with cut-off P-value 0.05. The backward selection includes three steps for selecting significant social variables, lifestyle variables, and health variables. In each step, the previous selected variables were forced in the model of the next step. For the models of suboptimal outcome at T2, sex, race, age at T1, and T1 HRQOL were forced in all models.

[†] Reference group: optimal individual HRQOL domains at T2
[§] Other: American Indian / Alaska Native, Asian or Pacific Islander, or unknown

© 2022 Schulte F et al. *JAMA Network Open*.

eTable 5. Predictive Factors for a Decline in HRQOL From Times 1 to 2 by 8 Domains of SF-36 and the Performance of Prediction Models

| Predictor | Time point | Level | A decline in PF from T1 to T2† | A decline in RP from T1 to T2† | A decline in BP from T1 to T2† | A decline in GH from T1 to T2† |
|---|---|---|---|---|---|---|
| | | | OR (95%CI; P-value) | OR (95%CI; P-value) | OR (95%CI; P-value) | OR (95%CI; P-value) |
| Age in years | T1 | | 1.06 (1.04 - 1.08; <.001) | 1.05 (1.03 - 1.07; <.001) | 0.99 (0.97 - 1.02; 0.70) | 0.99 (0.97 - 1.01; 0.50) |
| Sex | | Female | 1.95 (1.40 - 2.72; <.001) | 1.61 (1.23 - 2.11; <.001) | 2.31 (1.65 - 3.23; <.001) | 1.19 (0.92 - 1.53; 0.18) |
| | | Male | Reference | Reference | Reference | Reference |
| Race/ethnicity | | Black, non-Hispanic | 1.73 (0.70 - 4.29; 0.24) | 1.37 (0.62 - 3.06; 0.44) | 2.06 (0.90 - 4.72; 0.09) | 1.02 (0.44 - 2.37; 0.96) |
| | | Hispanic | 0.52 (0.17 - 1.55; 0.24) | 0.86 (0.39 - 1.90; 0.70) | 1.28 (0.56 - 2.96; 0.56) | 1.12 (0.55 - 2.28; 0.75) |
| | | Other | 0.57 (0.19 - 1.68; 0.31) | 1.27 (0.60 - 2.70; 0.53) | 1.40 (0.59 - 3.34; 0.45) | 1.56 (0.78 - 3.09; 0.21) |
| | | White, non-Hispanic | Reference | Reference | Reference | Reference |
| Years between T1 and T2 | | | 1.21 (0.98 - 1.50; 0.080) | 1.13 (0.94 - 1.35; 0.21) | 0.93 (0.74 - 1.18; 0.57) | 0.96 (0.80 - 1.15; 0.67) |
| Marital status | T1 | Widowed/divorced/ separated | | | | |
| | | Single | | | | |
| | | Married/Living with partner | | | | |
| Educational attainment | T1 | Less than high school | 2.99 (1.41 - 6.34; 0.004) | 1.83 (0.88 - 3.82; 0.11) | 5.23 (2.12 - 12.92; <.001) | |
| | | High school/GED | 1.55 (0.93 - 2.58; 0.09) | 1.44 (0.95 - 2.19; 0.08) | 1.69 (0.86 - 3.32; 0.13) | |
| | | Some college | 1.53 (1.10 - 2.13; 0.01) | 1.28 (0.96 - 1.69; 0.09) | 1.42 (0.91 - 2.23; 0.13) | |
| | | College graduate or postgraduate | Reference | Reference | Reference | |
| | T0 | Less than high school | | | 0.56 (0.28 - 1.14; 0.11) | |
| | | High school/GED | | | 1.03 (0.53 - 1.99; 0.94) | |
| | | Some college | | | 0.78 (0.47 - 1.29; 0.33) | |
| | | College graduate or postgraduate | | | Reference | |
| Employment status | T1 | Part-time | 0.98 (0.64 - 1.52; 0.94) | 0.84 (0.57 - 1.24; 0.39) | | |
| | | Unemployed | 1.48 (1.02 - 2.17; 0.04) | 1.60 (1.16 - 2.21; 0.004) | | |
| | | Full-time | Reference | Reference | | |
| Annual household income | T1 | <$20,000 | 1.56 (0.89 - 2.74; 0.12) | 1.75 (1.01 - 3.04; 0.05) | 1.05 (0.58 - 1.90; 0.88) | |
| | | $20,000 - $39,999 | 1.50 (0.95 - 2.37; 0.08) | 1.33 (0.92 - 1.93; 0.14) | 1.47 (0.95 - 2.27; 0.09) | |
| | | $40,000 - $59,999 | 1.29 (0.84 - 1.98; 0.25) | 0.85 (0.59 - 1.22; 0.37) | 0.78 (0.50 - 1.20; 0.26) | |
| | | $60,000 - $79,999 | 1.21 (0.74 - 1.98; 0.45) | 0.79 (0.53 - 1.20; 0.27) | 0.74 (0.45 - 1.24; 0.26) | |
| | | ≥$80,000 | Reference | Reference | Reference | |
| | T0 | <$20,000 | | | | 1.51 (0.96 - 2.37; 0.08) |
| | | $20,000 - $59,999 | | | | 1.08 (0.80 - 1.45; 0.62) |
| | | ≥$60,000 | | | | Reference |
| | From T0 to T1, focusing on available information | Worsen one category | | 0.94 (0.58 - 1.51; 0.80) | | 1.55 (1.03 - 2.32; 0.03) |
| | | Worsen more than one categories | | 0.16 (0.03 - 0.77; 0.02) | | 0.36 (0.10 - 1.31; 0.12) |
| | | Improved one category | | 0.84 (0.61 - 1.15; 0.27) | | 0.75 (0.55 - 1.04; 0.08) |
| | | Improved more than one categories | | 1.41 (0.66 - 3.01; 0.37) | | 0.69 (0.29 - 1.65; 0.40) |

© 2022 Schulte F et al. *JAMA Network Open*.

| | | | | | | |
|---|---|---|---|---|---|---|
| | | No changes | | Reference | | Reference |
| Living arrangement | T1 | Living dependently | 1.14 (0.75 - 1.72; 0.55) | | | 1.00 (0.72 - 1.40; 0.98) |
| | | Living independently | Reference | | | Reference |
| Primary care or oncology visits | T1 | No | 0.89 (0.65 - 1.22; 0.48) | 0.72 (0.55 - 0.93; 0.01) | | 0.83 (0.63 - 1.08; 0.17) |
| | | Yes (≥1 visits) | Reference | Reference | | Reference |
| | T0 | No | | | | 0.83 (0.64 - 1.07; 0.15) |
| | | Yes (≥1 visits) | | | | Reference |
| Cigarette smoking | T1 | Past smoker | | | 1.12 (0.72 - 1.74; 0.61) | |
| | | Current smoker | | | 1.15 (0.73 - 1.83; 0.55) | |
| | | Never smoker | | | Reference | |
| Physical activity | T1 | Inactive | 1.41 (1.05 - 1.90; 0.02) | | | |
| | | Active | Reference | | | |
| | T0 | Inactive | | | | 1.46 (1.09 - 1.96; 0.01) |
| | | Active | | | | Reference |
| Body weight status | T1 | Underweight/normal weight | | Reference | | |
| | | Overweight | | 1.21 (0.90 - 1.63; 0.20) | | |
| | | Obese | | 1.44 (1.05 - 1.98; 0.03) | | |
| | T0 | Underweight/normal weight | Reference | | Reference | Reference |
| | | Overweight | 1.12 (0.78 - 1.60; 0.54) | | 1.41 (0.98 - 2.02; 0.06) | 1.17 (0.87 - 1.58; 0.29) |
| | | Obese | 1.72 (1.12 - 2.64; 0.01) | | 1.44 (0.90 - 2.30; 0.13) | 2.26 (1.56 - 3.25; <.001) |
| Vision/eye disorders | T1 | Yes | | | 3.95 (1.47 - 10.62; 0.007) | |
| | | No | | | Reference | |
| | T0 | Yes | | | 0.31 (0.10 - 0.91; 0.03) | |
| | | No | | | Reference | |
| Hearing disorders | T1 | Yes | | 1.85 (1.15 - 2.96; 0.01) | | |
| | | No | | Reference | | |
| | T0 | Yes | 2.19 (1.25 - 3.87; 0.006) | | | |
| | | No | Reference | | | |
| Respiratory disorders | T1 | Yes | | 1.94 (1.30 - 2.88; 0.001) | 1.78 (1.10 - 2.89; 0.02) | 1.90 (1.23 - 2.94; 0.004) |
| | | No | | Reference | Reference | Reference |
| | T0 | Yes | 3.02 (1.91 - 4.78; <.001) | | | |
| | | No | Reference | | | |
| Cardiovascular disorders | T1 | Yes | 1.82 (1.32 - 2.51; <.001) | 1.82 (1.39 - 2.38; <.001) | 2.01 (1.31 - 3.07; 0.001) | 1.83 (1.39 - 2.40; <.001) |
| | | No | Reference | Reference | Reference | Reference |
| | T0 | Yes | | | 0.59 (0.34 - 1.04; 0.07) | |
| | | No | | | Reference | |
| Gastrointestinal disorders | T1 | Yes | | | | 1.59 (1.12 - 2.27; 0.009) |
| | | No | | | | Reference |
| | T0 | Yes | | | 1.64 (1.02 - 2.63; 0.04) | |
| | | No | | | Reference | |
| Musculoskeletal disorders | T1 | Yes | 2.54 (1.49 - 4.35; <.001) | 1.63 (1.04 - 2.55; 0.03) | | 7.06 (1.81 - 27.54; 0.005) |
| | | No | Reference | Reference | | Reference |
| | T0 | Yes | | | | 0.17 (0.04 - 0.71; 0.02) |
| | | No | | | | Reference |
| Neurologic disorders | T1 | Yes | 2.59 (1.78 - 3.77; <.001) | 2.37 (1.71 - 3.27; <.001) | 1.91 (1.28 - 2.85; 0.002) | 1.60 (1.13 - 2.27; 0.008) |
| | | No | Reference | Reference | Reference | Reference |
| Endocrinological disorders | T1 | Yes | | 1.49 (1.14 - 1.95; 0.004) | 2.02 (1.22 - 3.33; 0.006) | 1.62 (1.23 - 2.12; <.001) |
| | | No | | | | |

© 2022 Schulte F et al. *JAMA Network Open*.

| | | | | | | |
|---|---|---|---|---|---|---|
| | T0 | Yes | | | 0.52 (0.30 - 0.90; 0.02) | |
| | | No | | | Reference | |
| Depression | T1 | Yes | 1.81 (1.17 - 2.80; 0.008) | | 1.95 (1.21 - 3.12; 0.006) | |
| | | No | Reference | | Reference | |
| Somatization | T1 | Yes | | | | 1.87 (1.23 - 2.84; 0.003) |
| | | No | | | | Reference |
| Memory | T1 | Yes | | 1.71 (1.20 - 2.42; 0.003) | | |
| | | No | | Reference | | |
| Task efficiency | T1 | Yes | | | | 1.56 (1.15 - 2.11; 0.005) |
| | | No | | | | Reference |
| Emotional regulation | T1 | Yes | 1.79 (1.19 - 2.72; 0.006) | 1.82 (1.27 - 2.60; 0.001) | 1.91 (1.22 - 3.00; 0.005) | |
| | | No | Reference | Reference | Reference | |
| | | | | | | |
| **Model performance (area under the ROC curve)** | | | **Training dataset: 0.78; Test dataset: 0.75 (0.69-0.82)** | **Training dataset: 0.75; Test dataset: 0.75 (0.69-0.80)** | **Training dataset: 0.73; Test dataset: 0.65 (0.57-0.74)** | **Training dataset: 0.71; Test dataset: 0.63 (0.57-0.70)** |

© 2022 Schulte F et al. *JAMA Network Open*.

eTable 5. (Cont'd)

| Predictor | Time point | Level | A decline in VT from T1 to T2† | A decline in SF from T1 to T2† | A decline in RE from T1 to T2† | A decline in MH from T1 to T2† |
|---|---|---|---|---|---|---|
| | | | OR (95%CI; P-value) | OR (95%CI; P-value) | OR (95%CI; P-value) | OR (95%CI; P-value) |
| Age in years | T1 | | 0.99 (0.98 - 1.01; 0.58) | 1.01 (0.99 - 1.03; 0.48) | 1.03 (1.00 - 1.06; 0.05) | 0.99 (0.97 - 1.01; 0.36) |
| Sex | | Female | 1.87 (1.40 - 2.49; <.001) | 1.56 (1.19 - 2.04; 0.001) | 1.48 (1.06 - 2.06; 0.02) | 1.33 (0.99 - 1.79; 0.06) |
| | | Male | Reference | Reference | Reference | Reference |
| Race/ethnicity | | Black, non-Hispanic | 1.22 (0.45 - 3.26; 0.70) | 0.74 (0.26 - 2.10; 0.57) | 1.62 (0.67 - 3.89; 0.28) | 1.25 (0.50 - 3.09; 0.63) |
| | | Hispanic | 1.21 (0.57 - 2.56; 0.62) | 1.71 (0.90 - 3.24; 0.10) | 0.88 (0.36 - 2.16; 0.78) | 1.93 (1.00 - 3.70; 0.05) |
| | | Other§ | 0.88 (0.38 - 2.00; 0.75) | 1.23 (0.56 - 2.71; 0.61) | 1.08 (0.41 - 2.83; 0.88) | 0.73 (0.26 - 2.08; 0.56) |
| | | White, non-Hispanic | Reference | Reference | Reference | Reference |
| Years between T1 and T2 | | | 0.79 (0.64 - 0.97; 0.03) | 0.98 (0.80 - 1.18; 0.80) | 1.06 (0.85 - 1.32; 0.63) | 1.05 (0.85 - 1.29; 0.65) |
| Marital Status | T1 | Widowed/divorced/ separated | | | 1.87 (1.12 - 3.12; 0.02) | |
| | | Single | | | 1.18 (0.81 - 1.73; 0.39) | |
| | | Married/living with partner | | | Reference | |
| Educational attainment | T1 | Less than high school | | | 1.73 (0.71 - 4.21; 0.23) | |
| | | High school/GED | | | 1.07 (0.62 - 1.84; 0.82) | |
| | | Some college | | | 1.30 (0.92 - 1.82; 0.13) | |
| | | College graduate or postgraduate | | | Reference | |
| | From T0 to T1 | Improved | | | 1.34 (0.90 - 2.00; 0.15) | |
| | | No changes | | | Reference | |
| Employment status | T1 | Part-time | 0.69 (0.45 - 1.07; 0.10) | | 1.32 (0.85 - 2.04; 0.22) | 0.75 (0.47 - 1.18; 0.21) |
| | | Unemployed | 1.21 (0.86 - 1.70; 0.28) | | 1.91 (1.31 - 2.77; <.001) | 1.38 (0.98 - 1.94; 0.07) |
| | | Full-time | Reference | | Reference | Reference |
| Annual household income | T1 | <$20,000 | 1.92 (1.15 - 3.21; 0.01) | | | 1.65 (0.99 - 2.77; 0.06) |
| | | $20,000 - $39,999 | 1.56 (1.04 - 2.34; 0.03) | | | 1.11 (0.72 - 1.73; 0.63) |
| | | $40,000 - $59,999 | 1.37 (0.94 - 2.00; 0.10) | | | 1.14 (0.78 - 1.68; 0.49) |
| | | $60,000 - $79,999 | 1.08 (0.69 - 1.69; 0.75) | | | 0.93 (0.59 - 1.47; 0.76) |
| | | >$80,000 | Reference | | | Reference |
| | T0 | <$20,000 | | 1.52 (1.04 - 2.23; 0.03) | | |
| | | $20,000 - $59,999 | | 0.83 (0.62 - 1.11; 0.20) | | |
| | | >=$60,000 | | Reference | | |
| Living arrangement | T1 | Living dependently | | 1.28 (0.92 - 1.80; 0.15) | | 1.43 (1.00 - 2.03; 0.05) |
| | | Living independently | | Reference | | Reference |
| Health insurance | T0 | Uninsured | | | 1.45 (0.92 - 2.29; 0.11) | |
| | | Insured/Canadian resident | | | Reference | |
| | From T0 to T1 | Worsen | | 1.99 (1.28 - 3.09; 0.002) | | |
| | | Improved | | 1.24 (0.77 - 2.01; 0.38) | | |
| | | No changes | | Reference | | |
| Primary care or oncology visits | T1 | No | 0.71 (0.53 - 0.95; 0.02) | 0.77 (0.59 - 1.01; 0.06) | | |
| | | Yes (≥1 visits) | Reference | Reference | | |
| | From T0 to T1 | Worsen | | | | 1.30 (0.94 - 1.79; 0.11) |
| | | Improved | | | | 1.78 (1.18 - 2.69; 0.006) |

© 2022 Schulte F et al. *JAMA Network Open*.

| | | | | | | |
|---|---|---|---|---|---|---|
| | | No changes | | | | Reference |
| Cigarette smoking | T1 | Past smoker | 1.65 (1.15 - 2.36; 0.007) | 1.07 (0.73 - 1.55; 0.74) | | 1.21 (0.81 - 1.82; 0.35) |
| | | Current smoker | 1.55 (1.05 - 2.29; 0.03) | 1.60 (1.11 - 2.31; 0.01) | | 1.95 (1.32 - 2.89; <.001) |
| | | Never smoker | Reference | Reference | | Reference |
| Physical activity | T1 | Inactive | 1.59 (1.21 - 2.10; <.001) | | 1.54 (1.14 - 2.10; 0.005) | |
| | | Active | Reference | | Reference | |
| Hearing disorders | T1 | Yes | | 1.71 (1.03 - 2.84; 0.04) | | |
| | | No | | Reference | | |
| Respiratory disorders | T1 | Yes | 1.87 (1.20 - 2.90; 0.006) | | | 1.91 (1.22 - 2.98; 0.004) |
| | | No | Reference | | | Reference |
| Cardiovascular disorders | T1 | Yes | 1.68 (1.24 - 2.29; <.001) | 1.51 (1.13 - 2.02; 0.006) | | |
| | | No | Reference | Reference | | |
| Gastrointestinal disorders | T1 | Yes | 1.62 (1.12 - 2.36; 0.01) | 1.58 (1.12 - 2.24; 0.01) | | |
| | | No | Reference | Reference | | |
| Neurologic disorders | T1 | Yes | 1.53 (1.06 - 2.23; 0.03) | 1.59 (1.11 - 2.26; 0.01) | 2.01 (1.38 - 2.95; <.001) | |
| | | No | Reference | Reference | Reference | |
| Depression | T1 | Yes | 3.65 (2.29 - 5.82; <.001) | | 3.15 (1.89 - 5.24; <.001) | 3.02 (1.69 - 5.40; <.001) |
| | | No | Reference | | Reference | Reference |
| Somatization | T1 | Yes | 1.87 (1.18 - 2.97; 0.008) | 1.77 (1.19 - 2.63; 0.005) | 1.87 (1.20 - 2.91; 0.005) | |
| | | No | Reference | Reference | Reference | |
| Memory | T1 | Yes | | | | 1.55 (1.03 - 2.33; 0.04) |
| | | No | | | | Reference |
| Task efficiency | T1 | Yes | | | 2.20 (1.55 - 3.13; <.001) | |
| | | No | | | Reference | |
| Emotional regulation | T1 | Yes | 1.65 (1.05 - 2.58; 0.03) | 1.87 (1.29 - 2.73; 0.001) | | |
| | | No | Reference | Reference | | |
| | | | | | | |
| **Model performance (area under the ROC curve)** | | | **Training dataset: 0.73 Test dataset: 0.58 (0.51-0.66)** | **Training dataset: 0.68; test AUC 0.64 (0.58-0.71)** | **Training dataset: 0.75; test AUC 0.68 (0.59-0.76)** | **Training dataset: 0.67; test AUC 0.59 (0.52-0.66)** |

PF: physical functioning, RP: role limitation due to physical health problems, BP: bodily pain, GH: general health perceptions, VT: vitality, SF: social functioning, RE: role limitation due to emotional health problems, MH: mental health; GED = General educational development diploma; ROC = Receiver Operating Characteristic

Based on backward selection with cut-off P-value 0.05. The backward selection includes three steps for selecting significant social variables, lifestyle variables, and health variables. In each step, the previous selected variables were forced in the model of the next step. For the models of HRQOL changes from T1-T2, sex, race, age at T1, and years between T1 and T2 were forced in all models.

[†] Reference group: persistently optimal individual HRQOL domain from T1 to T2
[§] Other: American Indian / Alaska Native, Asian or Pacific Islander, or unknown

© 2022 Schulte F et al. *JAMA Network Open*.
